# Regioselective 1,4-trifluoromethylation of α,β-unsaturated ketones via a *S*-(trifluoromethyl)diphenylsulfonium salts/copper system

**DOI:** 10.3762/bjoc.9.257

**Published:** 2013-10-23

**Authors:** Satoshi Okusu, Yutaka Sugita, Etsuko Tokunaga, Norio Shibata

**Affiliations:** 1 Department of Frontier Materials, Graduate School of Engineering, Nagoya Institute of Technology, Gokiso, Showa-ku, Nagoya 466-8555, Japan

**Keywords:** 1,4-addition, copper, fluorine, Michael addition, organo-fluorine, trifluoromethylation

## Abstract

Regioselective conjugate 1,4-trifluoromethylation of α,β-unsaturated ketones by the use of shelf-stable electrophilic trifluoromethylating reagents, *S*-(trifluoromethyl)diphenylsulfonium salts and copper under mild conditions is described. A wide range of acyclic aryl–aryl–enones and aryl–alkyl–enones were converted into β-trifluoromethylated ketones in low to moderate yields.

## Introduction

**Scheme 1:**
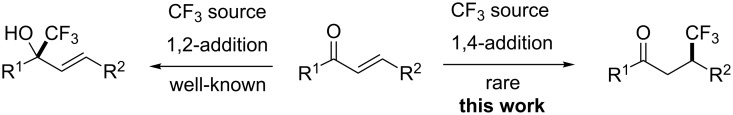
Trifluoromethylation of α,β-unsaturated ketones.

One of the challenges in synthetic organic chemistry is the nucleophilic 1,4-addition of the trifluoromethyl (CF_3_) group into electron-deficient internal alkenes as represented by the Michael addition reaction, even in a racemic, non-stereoselective fashion [1–5]. The nucleophilic trifluoromethylation to conjugated alkenes essentially occurs solely via a 1,2-addition [1–11], not a 1,4-addition (Scheme 1), with the exception of non-general examples of 1,4-additive trifluoromethylation of (trifluoromethyl)trimethylsilane (Me_3_SiCF_3_, Ruppert–Prakash reagent) to very specific substrates such as *trans*-1-benzoyl-2-(dimethylamino)ethylene [12], 2-polyfluoroalkylchromones [13–14], isoxazoles with a nitro group at the 4-position [15], and Morita–Baylis–Hillman adducts (via S_N_2’ [16] or successive S_N_2’/S_N_2’ mode [17]).

Sevenard and co-workers reported the nucleophilic 1,4-trifluoromethylation to chromones, coumarins and cyclohex-2-enone using the Ruppert–Prakash reagent, which was achieved by blocking the carbonyl moiety of the substrates with a bulky aluminium-centered Lewis acid with low to moderate yields [18]. Dilman and co-workers partially overcame this problem by using highly electrophilic alkenes bearing either Meldrum’ acids [19], or two geminal nitrile groups [20]. However, direct 1,4-trifluoromethylation to conventional α,β-unsaturated ketones such as chalcone is very tough, presumably due to the hardness of the CF_3_ anion. Recently, we reported the copper-mediated trifluoromethylation at the benzylic position by using shelf-stable electrophilic trifluoromethylating reagents, *S*-(trifluoromethyl)diphenylsulfonium salts, in good to high yields under mild conditions [21]. In this reaction, a bromide at the benzylic position would be replaced by a CF_3_ anion mediated by a copper via SET process, although the reaction mechanism is not clear. We envisaged that the system could be applicable to the conjugated 1,4-trifluoromethylation to simple chalcones. During the preparation of this article, the Nicewicz group showed a single example of conjugate trifluoromethylation of chalcone with sodium trifluoromethanesulfinate salt in the presence of *N*-methyl-9-mesitylacridinium as a photoredox catalyst resulting in a low product yield of 31% as a mixture of regioisomers (C2/C3 1.1:1) [22]. We disclose herein the regioselective 1,4-addition of the CF_3_ group into simple conjugated acyclic enones including chalcones using *S*-(trifluoromethyl)diphenylsulfonium salt **3** and a copper system in 11–37% yields (12 examples).

## Results and Discussion

We initiated our investigation with the reaction of chalcone (**1a**) using a series of electrophilic trifluoromethylating reagents **3** [23–26] in the presence of copper in DMF at 60 °C (Table 1), based on previously reported conditions [21]. First, the trifluoromethylation of **1a** with *S*-(trifluoromethyl)diphenylsulfonium salt **3a** was attempted, and a desired product **2a** was obtained in only 4% yield (Table 1, entry 1). Next the solvent was screened for yield improvement. We attempted the same reaction using NMP and DMSO, and the desired product **2a** was obtained in 6% and 11% yields (Table 1, entries 2 and 3, respectively). Interestingly, adding water (DMF/H_2_O 1:1) effectively improved the yield to 23% (Table 1, entry 4). Reactions mediated by other metals, such as Ni and Zn, either gave poor yields (Table 1, entries 5 and 6). The use of larger excesses of *S*-(trifluoromethyl)diphenylsulfonium salt **3a** (4.0 equiv) and Cu (6.0 equiv) in DMF/H_2_O (1:1) led to an increase in the yield of **2a** (Table 1, entry 9). The best result was obtained by treating **1a** at 60 °C in DMSO/H_2_O (1:1) in the presence of *S*-(trifluoromethyl)diphenylsulfonium salt **3a** (4.0 equiv) and Cu (6.0 equiv), leading to the isolation of **2a** in 37% yield (Table 1, entry 10). Using 4.0 equiv of Umemoto’s reagent **3b** instead of **3a** gave the product **2a** in 27% yield (Table 1, entry 12). *S*-(Trifluoromethyl)benzothiophenium salt **3c** [24], trifluoromethylsulfoxinium salt **3d** [25], and hypervalent iodine(III) CF_3_ reagent **3e** [26] did not proceed or provided only a trace amount of the desired product **2a** under the same reaction conditions (Table 1, entries 13–15). No reaction was observed using Ruppert–Prakash reagent in the presence of Cu under the same conditions (Table 1, entry 16). In all the cases, the reaction was regioselective and a trace amount of regioisomers and/or byproducts was detected in a crude mixture analyzed by ^19^F NMR. Under the best conditions shown in entry 10, we re-examined the reaction, but in the presence of TEMPO. The product formation was inhibited by TEMPO and *O*-trifluoromethylated TEMPO was detected in 2% by ^19^F NMR analysis (Table 1, entry 17).

**Table 1:** Optimization of CF_3_ reagents, metal, and solvent for copper-mediated conjugate trifluoromethylation of chalcone (**1a**).^a^

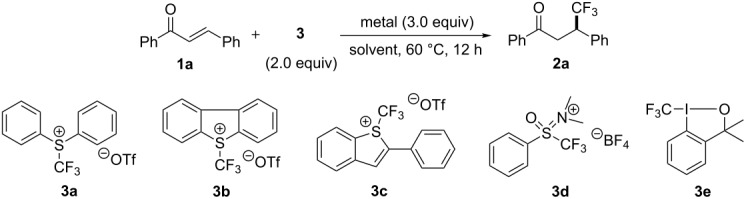				

Entry	CF_3_ reagent	metal	solvent	Yield (%)^b^

1	**3a**	Cu	DMF	4
2	**3a**	Cu	NMP	6
3	**3a**	Cu	DMSO	11
4	**3a**	Cu	DMF/H_2_O (1:1)	23
5	**3a**	Ni	DMF/H_2_O (1:1)	9
6	**3a**	Zn	DMF/H_2_O (1:1)	trace
7	**3a**	Cu	NMP/H_2_O (1:1)	trace
8	**3a**	Cu	DMSO/H_2_O (1:1)	5
9^c^	**3a**	Cu	DMF/H_2_O (1:1)	25
10^c^	**3a**	Cu	DMSO/H_2_O (1:1)	37
11	**3b**	Cu	DMSO/H_2_O (1:1)	24
12^c^	**3b**	Cu	DMSO/H_2_O (1:1)	27
13	**3c**	Cu	DMSO/H_2_O (1:1)	trace
14	**3d**	Cu	DMSO/H_2_O (1:1)	0
15	**3e**	Cu	DMSO/H_2_O (1:1)	trace
16	Me_3_SiCF_3_	Cu	DMSO/H_2_O (1:1)	0
17^c,d^	**3a**	Cu	DMSO/H_2_O (1:1)	trace^e^

^a^The reaction of **1a** with **3** (2.0 equiv) was carried out in the presence of metal (3.0 equiv) at 60 °C. ^b^Isolated yield. ^c^**3** (4.0 equiv) and metal (6.0 equiv) were used. ^d^The reaction was performed in the presence of TEMPO (4.0 equiv). ^e^*O*-Trifluoromethylated TEMPO was detected in 2% by ^19^F NMR.

With suitable conditions in hand, the scope of copper-mediated conjugate trifluoromethylation of α,β-unsaturated ketones **1** with **3a** was explored with a variety of substrates selected in order to establish the generality of the process (Table 2). With respect to the aryl ketone group, aromatic rings substituted with either electron-donating or -withdrawing substituents, such as methyl, methoxy, fluoro and chloro were tolerated (Table 2, entries 2–4). A heteroaromatic, furanyl-substituted enone was compatible with the same reaction conditions (Table 2, entry 6). We next examined substrates differing in the nature of the β-aryl substituents under the same reaction conditions. A series of compounds with aromatic rings substituted with either electron-donating or -withdrawing substituents, such as methyl, methoxy, fluoro and chloro were also acceptable. Furthermore, the β-alkyl-substituted enone also produced the desired product **2l** (Table 2, entry 12).

**Table 2:** Copper-mediated conjugate trifluoromethylation of α,β-unsaturated ketones **1** with **3a**.^a^

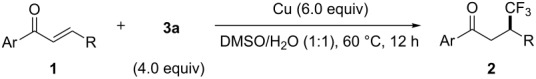					

Entry	**1**	Ar	R	**2**	Yield (%)^b^

1	**1a**	Ph	Ph	**2a**	37
2	**1b**	4-MeC_6_H_4_	Ph	**2b**	20
3	**1c**	4-MeOC_6_H_4_	Ph	**2c**	11
4	**1d**	4-FC_6_H_4_	Ph	**2d**	13
5	**1e**	4-ClC_6_H_4_	Ph	**2e**	22
6	**1f**	2-furanyl	Ph	**2f**	13
7	**1g**	Ph	4-MeC_6_H_4_	**2g**	12
8	**1h**	Ph	4-MeOC_6_H_4_	**2h**	12
9	**1i**	Ph	4-FC_6_H_4_	**2i**	17
10	**1j**	Ph	3-ClC_6_H_4_	**2j**	18
11	**1k**	Ph	4-ClC_6_H_4_	**2k**	13
12	**1l**	Ph	Me	**2l**	36

^a^The reaction of **1a** with **3a** (4.0 equiv) was carried out in the presence of Cu (6.0 equiv) at 60 °C. ^b^Isolated yield.

**Scheme 2:**
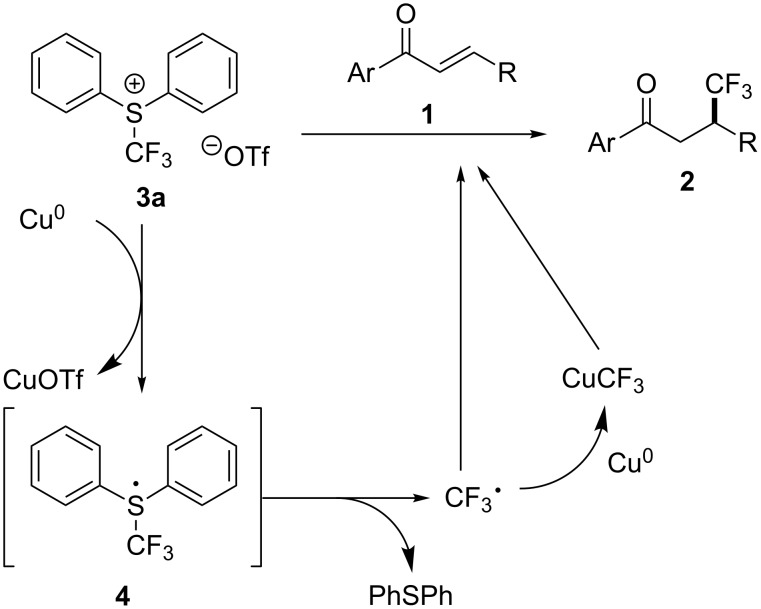
Proposed mechanism for the conjugate trifluoromethylation of α,β-unsaturated ketones by *S*-(trifluoromethyl)diphenylsulfonium salt and copper.

Based on these results, we hypothesized the reaction mechanism as shown in Scheme 2. First, the conjugate trifluoromethylation of α,β-unsaturated ketones would be initiated by a single-electron transfer between *S*-(trifluoromethyl)diphenylsulfonium salt **3a** and copper. The intermediate **4** decomposes to give the CF_3_ radical whose generation is supported by the TEMPO inhibition experiment. Ph_2_S was formed and checked by the ^1^H NMR spectroscopy. The resulting CF_3_ radical reacted directly with α,β-unsaturated ketones **1** and/or through the formation of CuCF_3_ species to provide the 1,4-adduct **2** in low to moderate yield. Although the true reactive species including CF_3_ radical and/or CuCF_3_ are not clear, the naked CF_3_ radical should be ruled out since high regioselectivity was observed, otherwise, a 1:1 mixture of regioisomers (C2/C3) should be detected like in the photoredox trifluoromethylation reaction [22].

## Conclusion

We developed for the first time the copper-mediated conjugate trifluoromethylation of simple α,β-unsaturated ketones through the use of shelf-stable electrophilic trifluoromethylating reagent **3a** under mild conditions. Although the yields are low, wide substrate generality was observed. Getting higher yields for this chemistry [27–29] and extension to asymmetric conjugate trifluoromethylation to simple α,β-unsaturated ketones are both our subsequent challenges and we are currently working in these directions.

## Supporting Information

File 1 Experimental section.
