# Supplementary material for: Regioselective 1,4-trifluoromethylation of α,β-unsaturated ketones via a S-(trifluoromethyl)diphenylsulfonium salts/copper system
Source: Beilstein J Org Chem. 2013 Oct 23;9:2189–93. doi: 10.3762/bjoc.9.257 (PMC3817478; doi:10.3762/bjoc.9.257)
Supplement: File 1 — Experimental section. [file Beilstein_J_Org_Chem-09-2189-s001.pdf]

Supporting Information

for

Regioselective 1,4-trifluoromethylation of α,β-unsaturated ketones via a *S*-(trifluoromethyl)diphenylsulfonium salts/copper system

Satoshi Okusu[1], Yutaka Sugita[1], Etsuko Tokunaga[1] and Norio Shibata*[1]

Address: [1] Department of Frontier Materials, Graduate School of Engineering, Nagoya Institute of Technology, Gokiso, Showa-ku, Nagoya 466-8555, Japan

Email: Norio Shibata* - nozshiba@nitech.ac.jp

* Corresponding author

Experimental section

General methods:

All reactions were performed in oven-dried glassware under a positive pressure of nitrogen. Solvents were transferred via syringe and were introduced into the reaction vessels though a rubber septum. All reactions were monitored by thin-layer chromatography (TLC) carried out on 0.25 mm Merck silica-gel (60-F_{254}). The TLC plates were visualized with UV light and 7% phosphomolybdic acid or $KMnO_4$ in water/heat. Column chromatography was carried out on a column packed with silica gel 60N spherical neutral size 63–210 μm. The ^{1}H-NMR (300 MHz), ^{19}F NMR (282 MHz), ^{13}C NMR (150.9 MHz) spectra in $CDCl_3$ were recorded on a Buruker Avance 600 and a Varian Mercury 300 spectrometer. Chemical shifts (δ) are expressed in ppm downfield from internal TMS or $CHCl_3$. The α,β-unsaturated ketones **1** were prepared according to literature. [1,2]

[1] A. Wilhelm, L. A. Lopez-Garcia, K. Busschots, W. Fröhner, F. Maurer, S. Boettcher, H. Zhang, J. O. Schulze, R. M. Biondi, M. Engel, *J. Med. Chem.* **2012**, *55*, 9817.
[2] B. A. Provencher, K. J. Bartelson, Y. Liu, B. M. Foxman, L. Deng, *Angew. Chem. Int. Ed.* **2011**, *50*, 10565.

General procedure for the copper mediated conjugate trifluoromethylation

A stirred solution of α,β-unsaturated ketones **1** (0.20 mmol), trifluoromethylsulfonium triflate **3a** (323 mg, 0.80 mmol, 4.0 equiv) and Cu (76.3 mg, 1.20 mmol, 6.0 equiv) in DMSO/H_2O (1:1, 1.0 mL) was heated at 60 °C for 12 h. After cooling down to room temperature, the reaction mixture was extracted with Et_2O, and the combined organic layers was washed with brine, dried over Na_2SO_4 and concentrated under reduced pressure. The residue was purified by column chromatography on silica gel to give β-trifluoromethylated ketone **2**.

4,4,4-Trifluoro-1,3-diphenyl-1-butanone (2a)[3]

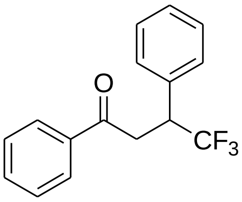

A reaction of **1a** (41.7 mg, 0.20 mmol), trifluoromethylsulfonium triflate **3a** (323 mg, 0.80 mmol, 4.0 equiv) and Cu (76.3 mg, 1.20 mmol, 6.0 equiv) in DMSO/H_2O (1:1, 1.0 mL) at 60 ºC for 12 h, and a purification by column chromatography on silica gel (*n*-hexane/benzene 8:2) to give β-trifluoromethylated ketone **2a** (10.2 mg, 37%) as a white solid.

^{1}H NMR ($CDCl_3$, 300 MHz) δ 3.60 (dd, *J* = 3.9, 17.4 Hz, 1H), 3.71 (ddd, *J* = 1.1, 8.3, 18.3 Hz, 1H), 4.19-4.32 (m, 1H), 7.26-7.49 (m, 7H), 7.55-7.61 (m, 1H), 7.93 (d, 8.4 Hz, 2H); ^{13}C NMR ($CDCl_3$, 150.9 MHz) δ 38.3, 44.8 (q, *J* = 27.2 Hz), 126.9 (q, *J* = 277.2 Hz), 128.02, 128.3, 128.68, 128.71, 129.0, 133.6, 134.6, 136.3, 195.3; ^{19}F NMR ($CDCl_3$, 282 MHz) δ -70.2 (d, *J* = 9.9 Hz, 3F); IR (KBr) 3347, 3068, 2959, 1966, 1682, 1595, 1500, 1450, 1433, 1357, 1306, 1254, 1162, 1106, 962, 880, 749, 704, 597, 517 cm^{-1}; mp = 67.0-68.0 ºC ($CHCl_3$); MS (EI, *m/z*) 278 (M^+), HRMS (EI) calcd. for $C_{16}H_{13}F_3O$ $(M)^+$: 278.0918 Found: 278.0929

4,4,4-Trifluoro-1-(4-methylphenyl)-3-phenyl-1-butanone (2b)

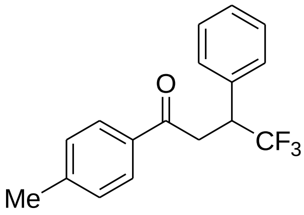

A reaction of **1b** (44.5 mg, 0.20 mmol), trifluoromethylsulfonium triflate **3a** (323 mg, 0.80 mmol, 4.0 equiv) and Cu (76.3 mg, 1.20 mmol, 6.0 equiv) in DMSO/H_2O (1:1, 1.0 mL) at 60 ºC for 12 h, and a purification by column chromatography on silica gel (*n*-hexane/benzene 8:2) to give

[3] A. Morigaki, T. Tanaka, T. Miyabe, T. Ishihara, T. Konno, *Org. Biomol. Chem.* **2013**, *11*, 586.

β-trifluoromethylated ketone **2b** (11.7 mg, 20%) as a white solid.

^{1}H NMR ($CDCl_3$, 300 MHz) δ 2.40, (s, 3H), 3.53-3.60 (m, 1H), 3.68 (ddd, *J* = 1.9, 8.9, 17.8 Hz, 1H), 4.21-4.28 (m, 1H), 7.24-7.38 (m, 7H), 7.81-7.84 (m, 2H); ^{13}C NMR ($CDCl_3$, 150.9 MHz) δ 21.6, 38.1, 44.8 (q, *J* = 27.7 Hz), 127.0 (q, *J* = 279.2 Hz), 128.1, 128.2, 128.6, 129.0, 129.4, 129.8, 133.8, 134.6, 144.5, 194.9; ^{19}F NMR ($CDCl_3$, 282 MHz) δ -70.1 (d, *J* = 8.7 Hz, 3F); IR (KBr) 3343, 3044, 2956, 2909, 1678, 1605, 1499, 1456, 1433, 1356, 1304, 1253, 1152, 1105, 962, 879, 821, 756, 700, 675, 591, 517 cm^{-1}; mp = 100.0-101.0 ºC ($CHCl_3$); MS (EI, *m/z*) 292 (M^+), HRMS (EI) calcd. for $C_{17}H_{15}F_3O$ $(M)^+$: 292.1075 Found: 292.1097

4,4,4-Trifluoro-1-(4-methoxyphenyl)-3-phenyl-1-butanone (2c)

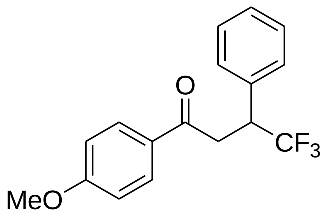

A reaction of **1c** (47.3 mg, 0.20 mmol), trifluoromethylsulfonium triflate **3a** (323 mg, 0.80 mmol, 4.0 equiv) and Cu (76.3 mg, 1.20 mmol, 6.0 equiv) in DMSO/H_2O (1:1, 1.0 mL) at 60 ºC for 12 h, and a purification by column chromatography on silica gel (*n*-hexane/benzene 8:2) to give β-trifluoromethylated ketone **2c** (6.7 mg, 11%) as a white solid.

^{1}H NMR ($CDCl_3$, 300 MHz) δ 3.53 (dd, *J* = 3.9, 17.4 Hz, 1H), 3.65 (dd, *J* = 9.0, 17.4 Hz, 1H), 3.86 (s, 3H), 4.18-4.31 (m, 1H), 6.92 (d, *J* = 8.4 Hz, 2H), 7.29-7.40 (m, 5H), 7.91 (d, *J* = 8.7 Hz, 2H); ^{13}C NMR ($CDCl_3$, 150.9 MHz) δ 37.8, 44.8 (q, *J* = 27.7 Hz), 55.5, 113.8, 127.0 (q, *J* = 283.7 Hz), 128.2, 128.6, 129.0, 129.4, 130.3, 134.7, 163.8, 193.7; ^{19}F NMR ($CDCl_3$, 282 MHz) δ -70.1 (d, *J* = 8.7 Hz, 3F); IR (KBr) 3331, 3044, 2977, 2941, 2909, 2845, 2582, 1673, 1604, 1573, 1512, 1423, 1305, 1258, 1149, 1105, 1032, 961, 848, 757, 700, 595 cm^{-1}; mp = 86.5-87.5 ºC ($CHCl_3$); MS (EI, *m/z*) 308 (M^+), HRMS (EI) calcd. for $C_{17}H_{15}F_3O_2$ $(M)^+$: 308.1024 Found: 308.1043

4,4,4-Trifluoro-1-(4-fluorophenyl)-3-phenyl-1-butanone (2d)

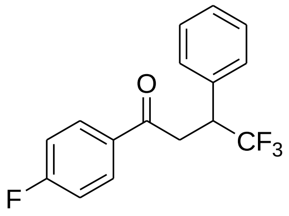

A reaction of **1d** (45.3 mg, 0.20 mmol), trifluoromethylsulfonium triflate **3a** (323 mg, 0.80 mmol, 4.0 equiv) and Cu (76.3 mg, 1.20 mmol, 6.0 equiv) in DMSO/H_2O (1:1, 1.0 mL) at 60 ºC for 12 h, and a purification by column chromatography on silica gel (*n*-hexane/benzene 8:2) to give

β-trifluoromethylated ketone **2d** (7.9 mg, 13%) as a white solid.

^{1}H NMR ($CDCl_3$, 300 MHz) δ 3.47-3.59 (m, 1H), 3.62-3.71 (m, 1H), 4.23 (m, 1H), 7.10-7.15 (m, 2H), 7.32-7.37 (m, 5H), 7.95 (m, 2H); ^{13}C NMR ($CDCl_3$, 150.9 MHz) δ 38.2, 44.8 (q, *J* = 27.7 Hz), 115.9 (d, 22.6 Hz), 126.9 (q, *J* = 278.7 Hz), 128.3, 128.7, 129.0, 130.7 (d, 9.1 Hz), 132.7 (d, 3.0 Hz), 134.5, 166.0 (d, 255.0 Hz), 193.7; ^{19}F NMR ($CDCl_3$, 282 MHz) δ -70.2 (d, *J* = 9.0 Hz, 3F), 104.7 (s, 1H); IR (KBr) 3363, 3074, 3033, 2946, 2343, 1688, 1600, 1508, 1451, 1432, 1375, 1306, 1243, 1164, 1101, 991, 878, 841, 702, 672, 625, 588, 516, 436 cm^{-1}; mp = 68.5-69.5 ºC ($CHCl_3$); MS (EI, *m/z*) 296 (M^+), HRMS (EI) calcd. for $C_{16}H_{12}F_4O$ $(M)^+$: 296.0824 Found: 296.0845

1-(4-Chlorophenyl)-4,4,4-trifluoro-3-phenyl-1-butanone (2e)

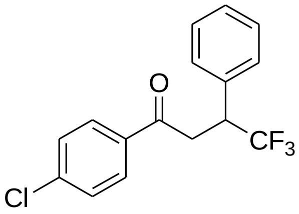

A reaction of **1e** (48.5 mg, 0.20 mmol), trifluoromethylsulfonium triflate **3a** (323 mg, 0.80 mmol, 4.0 equiv) and Cu (76.3 mg, 1.20 mmol, 6.0 equiv) in DMSO/H_2O (1:1, 1.0 mL) at 60 ºC for 12 h, and a purification by column chromatography on silica gel (*n*-hexane/benzene 8:2) to give β-trifluoromethylated ketone **2e** (10.9 mg, 22%) as a white solid.

^{1}H NMR ($CDCl_3$, 300 MHz) δ 3.56 (dd, *J* = 5.3, 17.6 Hz, 1H), 3.65-3.71 (m, 1H), 4.16-4.30 (m, 1H), 7.26-7.45 (m, 7H), 7.85-7.88 (m, 2H); ^{13}C NMR ($CDCl_3$, 150.9 MHz) δ 38.2, 44.8 (q, *J* = 27.7 Hz), 126.9 (q, *J* = 275.6 Hz), 128.4, 128.7, 128.96, 129.04, 129.4, 134.4, 134.6, 140.1, 194.1; ^{19}F NMR ($CDCl_3$, 282 MHz) δ -70.2 (d, *J* = 9.9 Hz, 3F); IR (KBr) 3358, 3096, 3066, 3036, 2937, 1686, 1590, 1490, 1455, 1402, 1303, 1246, 1222, 1164, 1105, 987, 877, 833, 754, 699, 665, 620, 458 cm^{-1}; mp = 74.0-75.0 ºC ($CHCl_3$); MS (EI, *m/z*) 312 (M^+), HRMS (EI) calcd. for $C_{16}H_{12}ClF_3O$ $(M)^+$: 312.0529 Found: 312.0524

4,4,4-Trifluoro-1-(2-furanyl)-3-phenyl-1-butanone (2f)

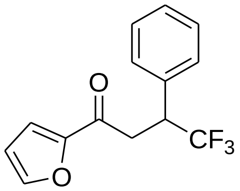

A reaction of **1f** (39.6 mg, 0.20 mmol), trifluoromethylsulfonium triflate **3a** (323 mg, 0.80 mmol, 4.0 equiv) and Cu (76.3 mg, 1.20 mmol, 6.0 equiv) in DMSO/H_2O (1:/1, 1.0 mL) at 60 ºC for 12 h, and a purification by column chromatography on silica gel (*n*-hexane/benzene 8:2) to give

β-trifluoromethylated ketone **2f** (7.2 mg, 13%) as a white solid.

^{1}H NMR ($CDCl_3$, 300 MHz) δ 3.45 (dd, *J* = 4.8, 17.4 Hz, 1H), 3.56 (dd, *J* = 8.7, 17.4 Hz, 1H), 4.16-4.24 (m, 1H), 6.52-6.54 (m, 1H), 7.18-7.19 (m, 1H), 7.26-7.37 (m, 5H), 7.58 (s, 1H); ^{13}C NMR ($CDCl_3$, 150.9 MHz) δ 38.0, 44.4 (q, *J* = 27.7 Hz), 112.5, 117.5, 126.8 (q, *J* = 277.2 Hz), 128.3, 128.7, 129.0, 134.2, 146.7, 152.2, 184.6; ^{19}F NMR ($CDCl_3$, 282 MHz) δ -70.3 (d, *J* = 9.9 Hz, 3F); IR (KBr) 3316, 3099, 3065, 3046, 2960, 2924, 2856, 2727, 2643, 2547, 2467, 2358, 2113, 1965, 1901, 1500, 843, 800, 541, 455 cm^{-1}; mp = 84.5-85.5 ºC ($CHCl_3$); MS (EI, *m/z*) 268 (M^+), HRMS (EI) calcd. for $C_{14}H_{11}F_3O_2$ $(M)^+$: 268.0711 Found: 268.0688

4,4,4-Trifluoro-3-(4- methylphenyl)-1-phenyl-1-butanone (2g)[3]

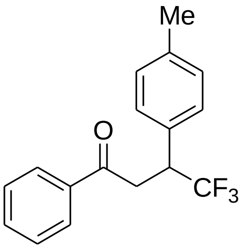

A reaction of **1g** (44.5 mg, 0.20 mmol), trifluoromethylsulfonium triflate **3a** (323 mg, 0.80 mmol, 4.0 equiv) and Cu (76.3 mg, 1.20 mmol, 6.0 equiv) in DMSO/H_2O (1:1, 1.0 mL) at 60 ºC for 12 h, and a purification by column chromatography on silica gel (*n*-hexane/benzene 8:2) to give β-trifluoromethylated ketone **2g** (6.9 mg, 12%) as a white solid.

^{1}H NMR ($CDCl_3$, 300 MHz) δ 2.31, (s, 3H), 3.55-3.61 (m, 1H), 3.69 (dd, *J* = 9.2, 17.6 Hz, 1H), 4.18-4.21 (m, 1H), 7.14 (d, *J* = 7.2 Hz, 2H), 7.28 (d, *J* = 7.8 Hz, 2H), 7.45 (t, *J* = 7.2 Hz, 2H), 7.55-7.57 (m, 1H), 7.93 (d, 7.5 Hz, 2H); ^{13}C NMR ($CDCl_3$, 150.9 MHz) δ 21.1, 38.2, 44.4 (q, *J* = 28.7 Hz), 127.1 (q, *J* = 284.7 Hz), 128.0, 128.7, 128.8, 129.4, 131.5, 133.5, 136.3, 138.1, 195.4; ^{19}F NMR ($CDCl_3$, 282 MHz) δ -70.3 (d, *J* = 9.9 Hz, 3F); IR (KBr) 3347, 3040, 2921, 1683, 1595, 1519, 1450, 1428, 1306, 1254, 1186, 1150, 1098, 1000, 885, 809, 765, 742, 685, 632, 599, 511 cm^{-1}; mp = 94.5-95.0 ºC ($CHCl_3$); MS (EI, *m/z*) 292 (M^+), MS (EI, *m/z*) 292 (M^+), HRMS (EI) calcd. for $C_{17}H_{15}F_3O$ $(M)^+$: 292.1075 Found: 292.1074

4,4,4-Trifluoro-3-(4-methoxyphenyl)-1-phenyl-1-butanone (2h)[3]

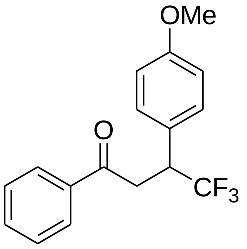

A reaction of **1h** (47.7 mg, 0.20 mmol), trifluoromethylsulfonium triflate **3a** (323 mg, 0.80 mmol, 4.0 equiv) and Cu (76.3 mg, 1.20 mmol, 6.0 equiv) in DMSO/H_2O (1:1, 1.0 mL) at 60 ºC for 12 h, and a purification by column chromatography on silica gel (*n*-hexane/benzene 8:2) to give β-trifluoromethylated ketone **2h** (7.4 mg, 12%) as a white solid.

^{1}H NMR ($CDCl_3$, 300 MHz) δ 3.56 (dd, *J* = 4.1, 17.6 Hz, 1H), 3.67 (dd, *J* = 9.0, 17.4 Hz, 1H), 3.77 (s, 3H), 4.13-4.26 (m, 1H), 6.86 (d, 8.4 Hz, 2H), 7.31 (d, *J* = 8.1 Hz, 2H), 7.46 (t, *J* = 7.5 Hz, 2H), 7.57 (t, *J* = 7.4 Hz, 1H), 7.93 (d, *J* = 8.1 Hz, 2H); ^{13}C NMR ($CDCl_3$, 150.9 MHz) δ 38.3, 44.0 (q, *J* = 27.7 Hz), 55.2, 114.1, 126.5, 127.0 (q, *J* = 279.2 Hz), 128.0, 128.7, 130.1, 133.5, 136.3, 159.4, 195.4; ^{19}F NMR ($CDCl_3$, 282 MHz) δ -70.5 (d, *J* = 9.9 Hz, 3F); IR (KBr) 3005, 2961, 2840, 1684, 1614, 1518, 1449, 1431, 1307, 1245, 1181, 1163, 1097, 1034, 962, 884, 819, 765, 685, 598, 531 cm^{-1}; mp = 89.0-90.0 ºC ($CHCl_3$); MS (EI, *m/z*) 308 (M^+), HRMS (EI) calcd. for $C_{17}H_{15}F_3O_2$ $(M)^+$: 308.1024 Found: 308.1048

4,4,4-Trifluoro-3-(4-fluorophenyl)-1-phenyl-1-butanone (2i)

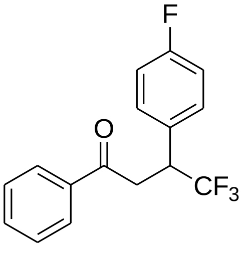

A reaction of **1i** (45.3 mg, 0.20 mmol), trifluoromethylsulfonium triflate **3a** (323 mg, 0.80 mmol, 4.0 equiv) and Cu (76.3 mg, 1.20 mmol, 6.0 equiv) in DMSO/H_2O (1:1, 1.0 mL) at 60 ºC for 12 h, and a purification by column chromatography on silica gel (*n*-hexane/benzene 8:2) to give β-trifluoromethylated ketone **2i** (10.1 mg, 17%) as a white solid.

^{1}H NMR ($CDCl_3$, 300 MHz) δ 3.59 (dd, *J* = 4.5, 17.6 Hz, 1H), 3.66-3.72 (m, 1H), 4.17-4.31 (m, 1H), 7.00-7.06 (m, 2H), 7.37 (t, *J* = 6.5 Hz, 2H), 7.44-7.49 (m, 2H), 7.57-7.61 (m, 1H), 7.92 (d, 8.4 Hz, 2H); ^{13}C NMR ($CDCl_3$, 150.9 MHz) δ 38.3, 44.1 (q, *J* = 27.7 Hz), 115.7 (d, *J* = 21.1 Hz), 126.8 (q, J = 279.7 Hz), 128.0, 128.8, 130.3, 130.7 (d, J = 7.5 Hz), 133.7, 136.2, 162.6 (d, *J* = 247.5 Hz), 195.1; ^{19}F NMR ($CDCl_3$, 282 MHz) δ -70.4 (d, *J* = 9.9 Hz, 3F), -114.3 (s, 1F); IR (KBr) 3351, 3060, 2925,

1687, 1596, 1518, 1450, 1427, 1308, 1257, 1167, 1149, 1112, 961, 922, 890, 828, 744, 722, 686, 598, 525, 492 cm^{-1}; mp = 97.5-98.5 °C ($CHCl_3$); MS (EI, *m/z*) 296 (M^+), HRMS (EI) calcd. for $C_{16}H_{12}F_4O$ $(M)^+$: 296.0824 Found: 296.0842

3-(3-Chlorophenyl)-4,4,4-trifluoro-1-phenyl-1-butanone (2j)[3]

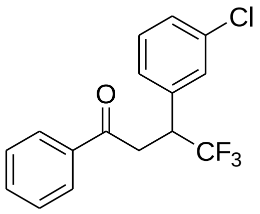

A reaction of **1j** (48.4 mg, 0.20 mmol), trifluoromethylsulfonium triflate **3a** (323 mg, 0.80 mmol, 4.0 equiv) and Cu (76.3 mg, 1.20 mmol, 6.0 equiv) in DMSO/H_2O (1:1, 1.0 mL) at 60 °C for 12 h, and a purification by column chromatography on silica gel (*n*-hexane/benzene 8:2) to give β-trifluoromethylated ketone **2j** (11.5 mg, 18%) as a colorless oil.

^{1}H NMR ($CDCl_3$, 300 MHz) δ 3.58-3.72 (m, 2H), 4.22-4.27 (m, 1H), 7.27-7.29 (m, 3H), 74.0 (s, 1H), 7.47 (t, J = 6.9 Hz, 2H), 7.57-7.62 (m, 1H), 7.92-7.95 (m, 2H); ^{13}C NMR ($CDCl_3$, 150.9 MHz) δ 38.2, 44.5 (q, J = 27.7 Hz), 126.6 (q, J = 279.7 Hz), 127.4, 128.0, 128.6, 128.8, 129.1, 129.9, 133.7, 134.5, 136.0, 136.5, 194.9; ^{19}F NMR ($CDCl_3$, 282 MHz) δ -70.0 (d, J = 9.0 Hz, 3F); IR (neat) 3065, 2921, 1692, 1598, 1577, 1478, 1449, 1301, 1256, 1158, 1109, 1002, 908, 782, 755, 688, 608, 496, 486, 468, 458, 428 cm^{-1}; MS (EI, *m/z*) 312 (M^+), HRMS (EI) calcd. for $C_{16}H_{12}ClF_3O$ $(M)^+$: 312.0529 Found: 312.0558

3-(4-Chlorophenyl)-4,4,4-trifluoro-1-phenyl-1-butanone (2k)[3]

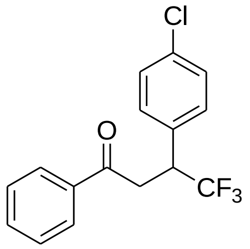

A reaction of **1k** (48.4 mg, 0.20 mmol), trifluoromethylsulfonium triflate **3a** (323 mg, 0.80 mmol, 4.0 equiv) and Cu (76.3 mg, 1.20 mmol, 6.0 equiv) in DMSO/H_2O (1:1, 1.0 mL) at 60 °C for 12 h, and a purification by column chromatography on silica gel (*n*-hexane/benzene 8:2) to give β-trifluoromethylated ketone **2k** (7.9 mg, 13%) as a white solid.

^{1}H NMR ($CDCl_3$, 300 MHz) δ 3.56-3.66 (m, 2H), 4.20-4.24 (m, 1H), 7.26-7.33 (m, 4H), 7.47 (t, J = 6.9 Hz, 2H), 7.59 (t, J = 6.6 Hz, 1H), 7.92 (d, J = 7.5 Hz, 2H); ^{13}C NMR ($CDCl_3$, 150.9 MHz) δ 38.1, 44.3 (q, J = 27.2 Hz), 126.6 (q, J = 272.1 Hz), 128.0, 128.8, 128.9, 130.3, 133.0, 133.7, 134.3, 136.1,

195.0; ^{19}F NMR ($CDCl_3$, 282 MHz) δ -70.2 (d, *J* = 9.9 Hz, 3F); IR (KBr) 3347, 3064, 2923, 1685, 1595, 1496, 1450, 1426, 1308, 1250, 1154, 1101, 1015, 888, 823, 778, 753, 728, 685, 625, 596, 518, 425 cm^{-1}; mp = 101.0-102.0 ºC ($CHCl_3$); MS (EI, *m/z*) 312 (M^+), HRMS (EI) calcd. for $C_{16}H_{12}ClF_3O$ $(M)^+$: 312.0529 Found: 312.0544

4,4,4-Trifluoro-3-methyl-1-phenyl-1-butanone (2l)[4]

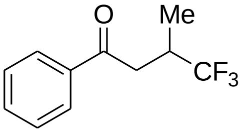

A reaction of **1l** (29.2 mg, 0.20 mmol), trifluoromethylsulfonium triflate **3a** (323 mg, 0.80 mmol, 4.0 equiv) and Cu (76.3 mg, 1.20 mmol, 6.0 equiv) in DMSO/H_2O (1:1, 1.0 mL) at 60 ºC for 12 h, and a purification by column chromatography on silica gel (*n*-hexane/benzene 8:2) to give β-trifluoromethylated ketone **2l** (15.4 mg, 36%) as a colorless oil.

^{1}H NMR ($CDCl_3$, 300 MHz) δ 1.19 (d, J = 5.7 Hz, 3H), 3.01-3.05 (m, 2H), 3.29-3.33 (m, 1H), 7.47-7.61 (m, 3H), 7.97 (d, *J* = 7.8 Hz, 2H); ^{13}C NMR ($CDCl_3$, 150.9 MHz) δ 13.2 (d, J = 1.5 Hz), 33.9 (q, *J* = 27.7 Hz), 38.4 (d, 1.5 Hz), 128.0, 128.3 (q, *J* = 278.7 Hz), 128.7, 133.5, 136.4, 196.3; ^{19}F NMR ($CDCl_3$, 282 MHz) δ -74.0 (d, *J* = 7.9 Hz, 3F); IR (neat) 2988, 2950, 1779, 1690, 1598, 1581, 1449, 1387, 1347, 1301, 1268, 1216, 1173, 1127, 1081, 1022, 992, 920, 754, 689, 624, 501, 460 cm^{-1}; MS (EI, *m/z*) 216 (M^+), HRMS (EI) calcd. for $C_{11}H_{11}F_3O$ $(M)^+$: 216.0762 Found: 216.0743

[4] V. Bizet, X. Pannecoucke, J.-L. Renaud, D. Cahard, *Angew. Chem. Int. Ed.* **2012**, *51*, 6467.

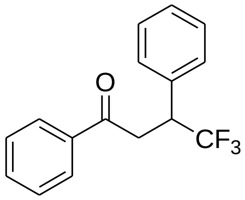

2a

^{1}H-NMR

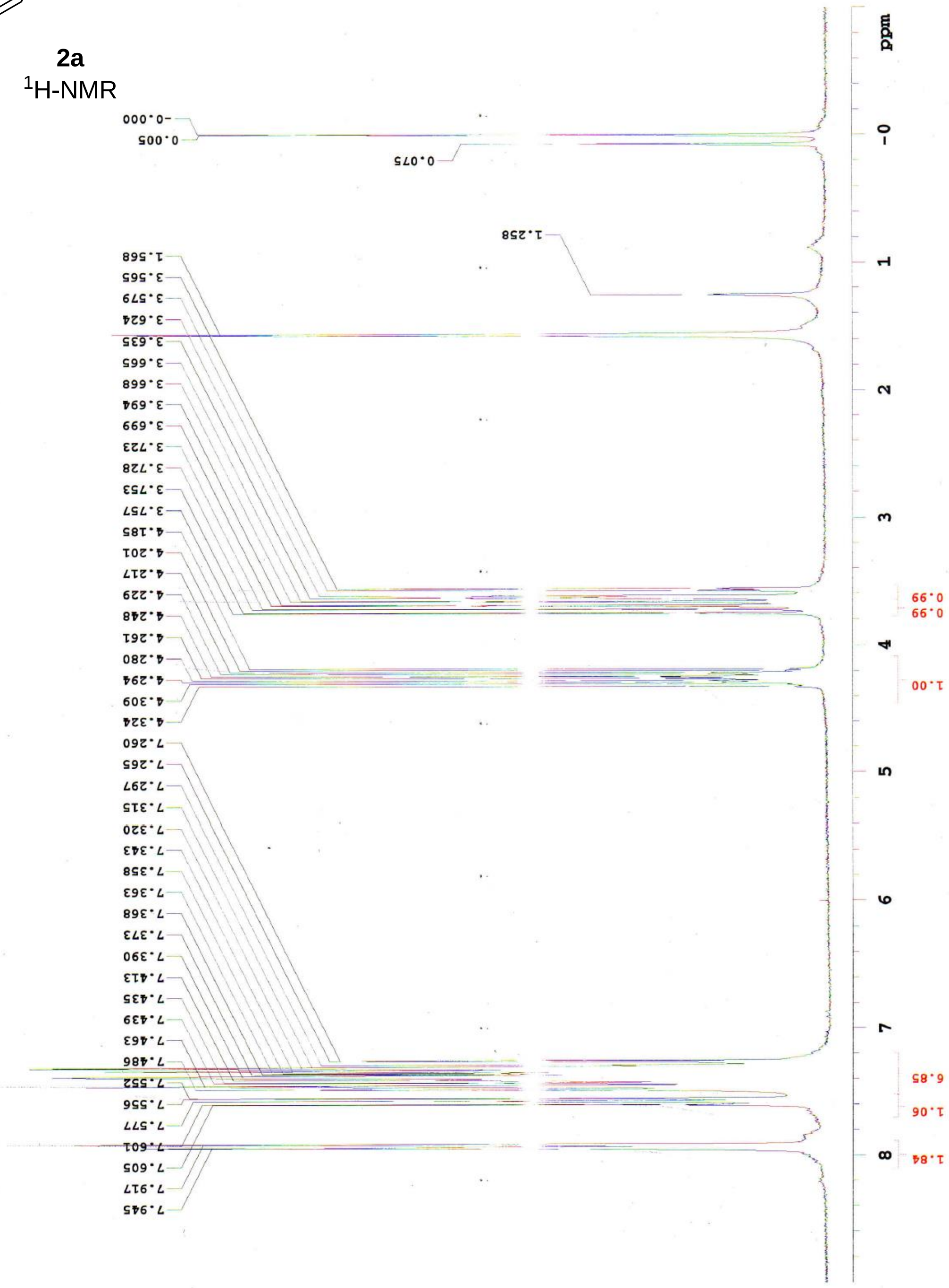
-0.000
0.005
0.075
1.258
1.568
3.565
3.579
3.624
3.635
3.665
3.668
3.694
3.699
3.723
3.728
3.753
3.757
4.185
4.201
4.217
4.229
4.248
4.261
4.280
4.294
4.309
4.324
7.260
7.265
7.297
7.315
7.320
7.343
7.358
7.363
7.368
7.373
7.390
7.413
7.435
7.439
7.463
7.486
7.552
7.556
7.577
7.601
7.605
7.917
7.945
0.99
0.99
1.00
6.85
1.06
1.84
ppm
-0
1
2
3
4
5
6
7
8

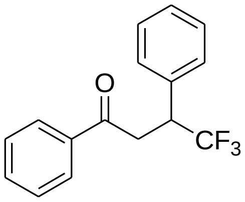

2a

^{19}F-NMR

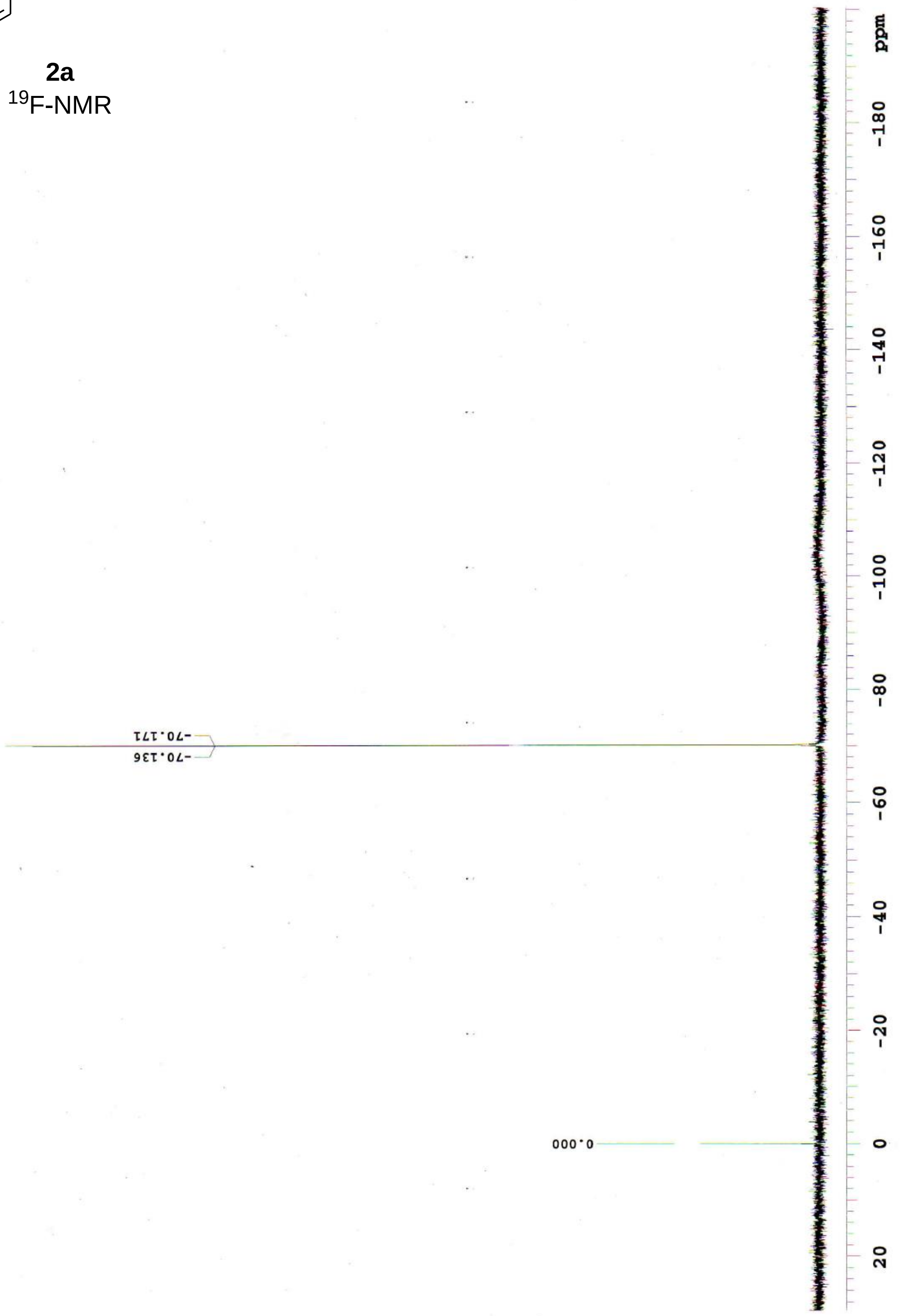

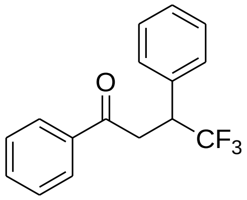
O
CF3

2a

^{13}C-NMR

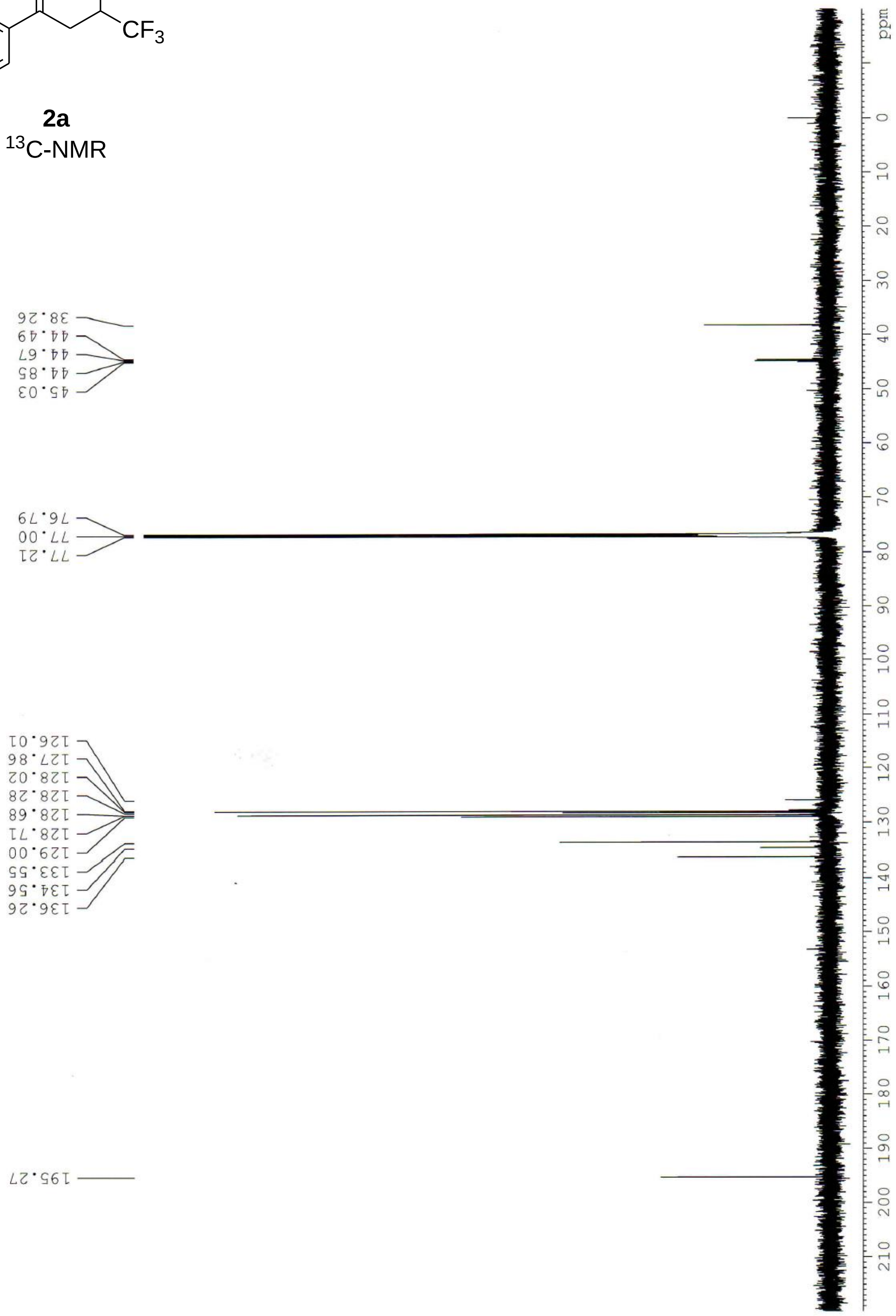
38.26
44.49
44.67
44.85
45.03
76.79
77.00
77.21
126.01
127.86
128.02
128.28
128.68
128.71
129.00
133.55
134.56
136.26
195.27
ppm

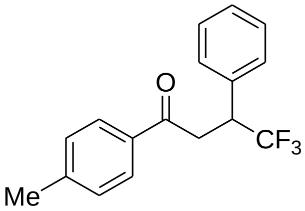

2b

^{1}H-NMR

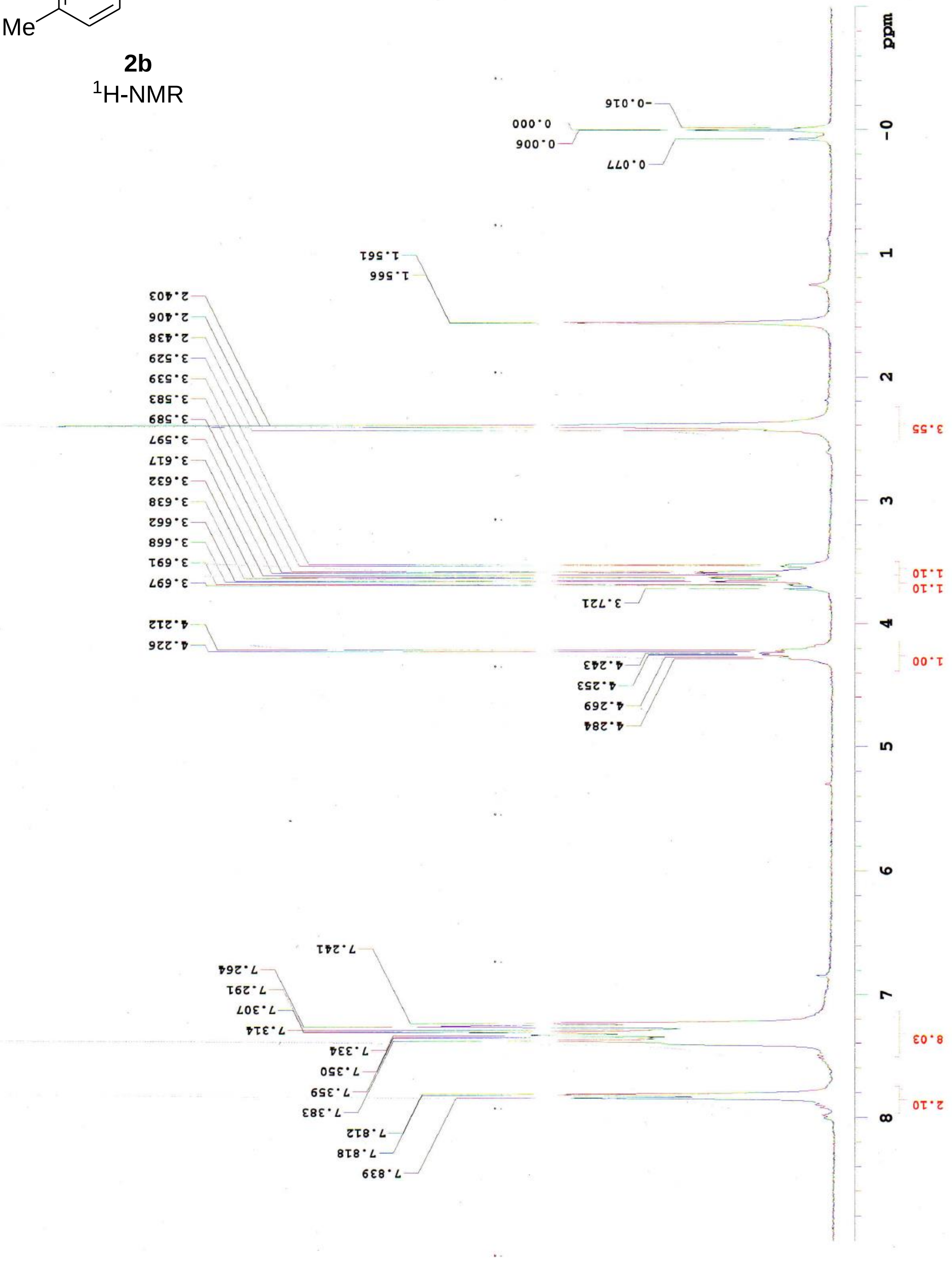

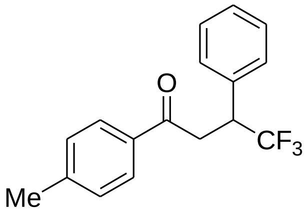

2b

^{19}F-NMR

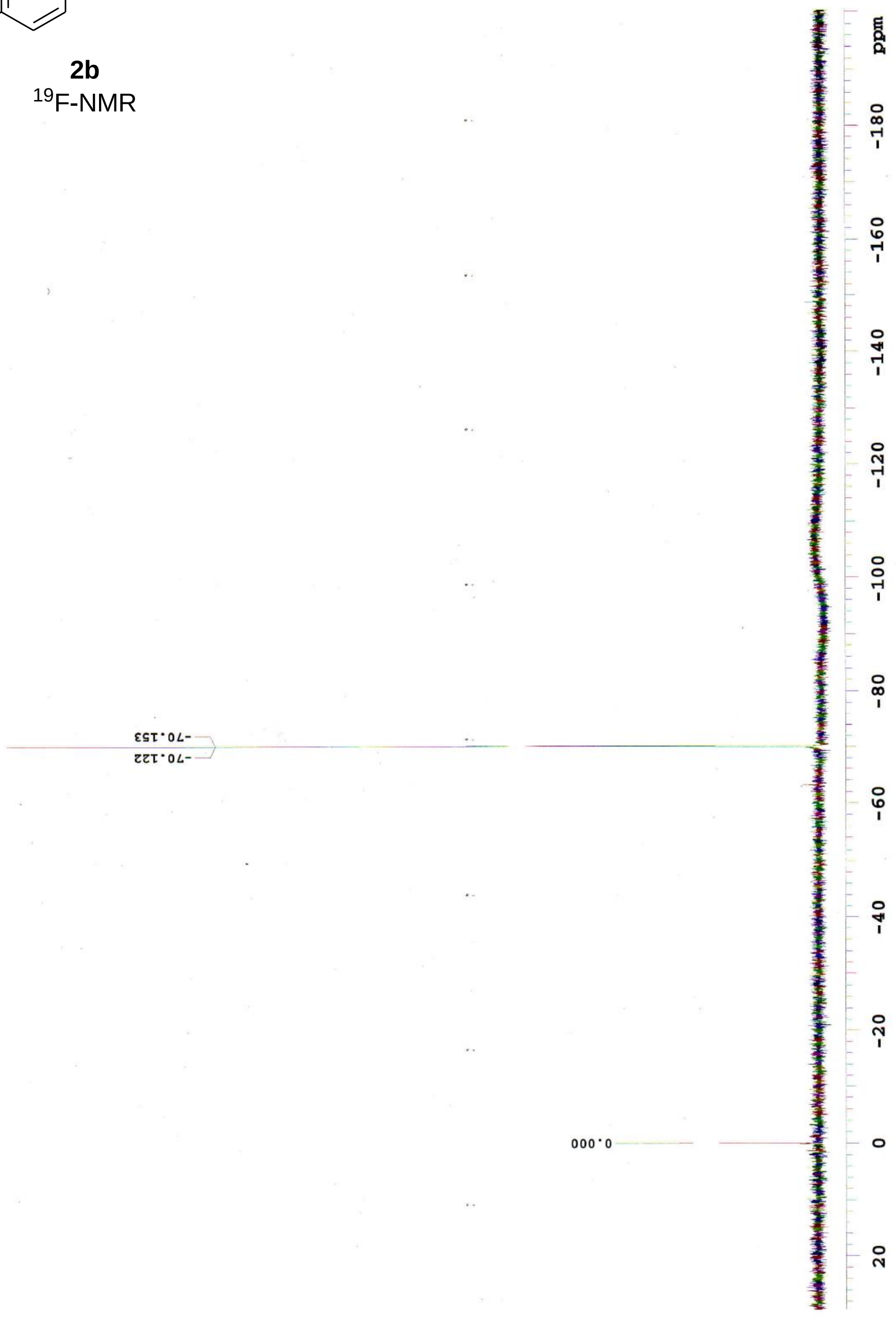

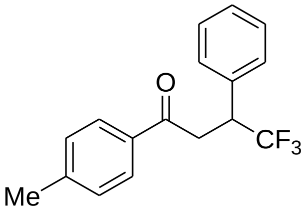
O
CF3
Me

2b

^{13}C-NMR

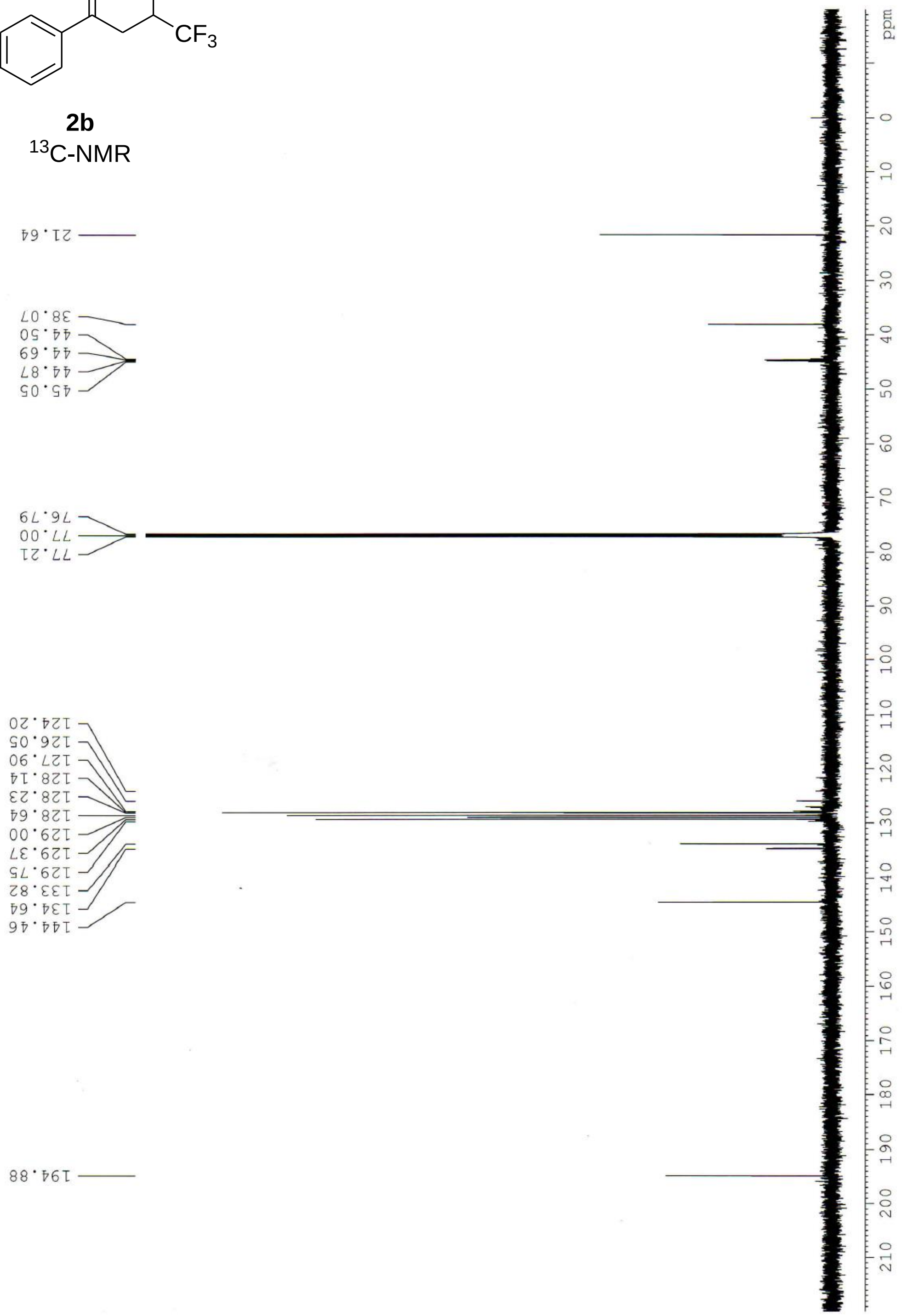
21.64
38.07
44.50
44.69
44.87
45.05
76.79
77.00
77.21
124.20
126.05
127.90
128.14
128.23
128.64
129.00
129.37
129.75
133.82
134.64
144.46
194.88
ppm

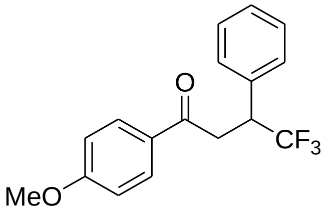
O
CF3
MeO

2c
^{1}H-NMR

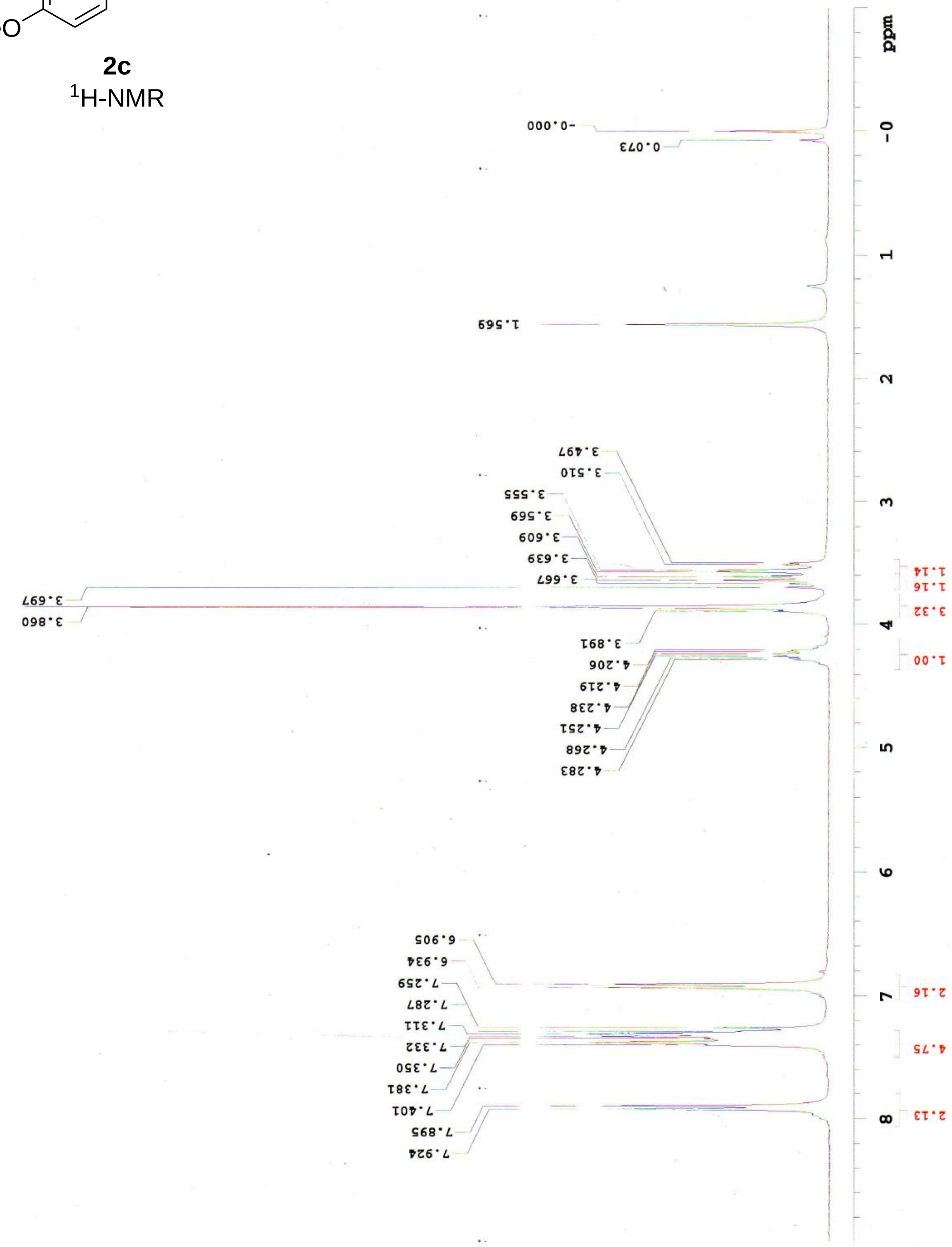
ppm
-0.000
0.073
1.569
3.497
3.510
3.555
3.569
3.609
3.639
3.667
3.697
3.860
3.891
4.206
4.219
4.238
4.251
4.268
4.283
6.905
6.934
7.259
7.287
7.311
7.332
7.350
7.381
7.401
7.895
7.924
1.14
1.16
3.32
1.00
2.16
4.75
2.13

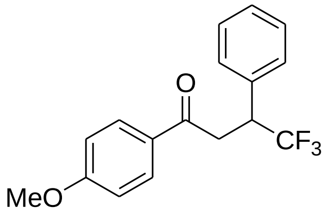
O
CF3
MeO

2c
^{19}F-NMR

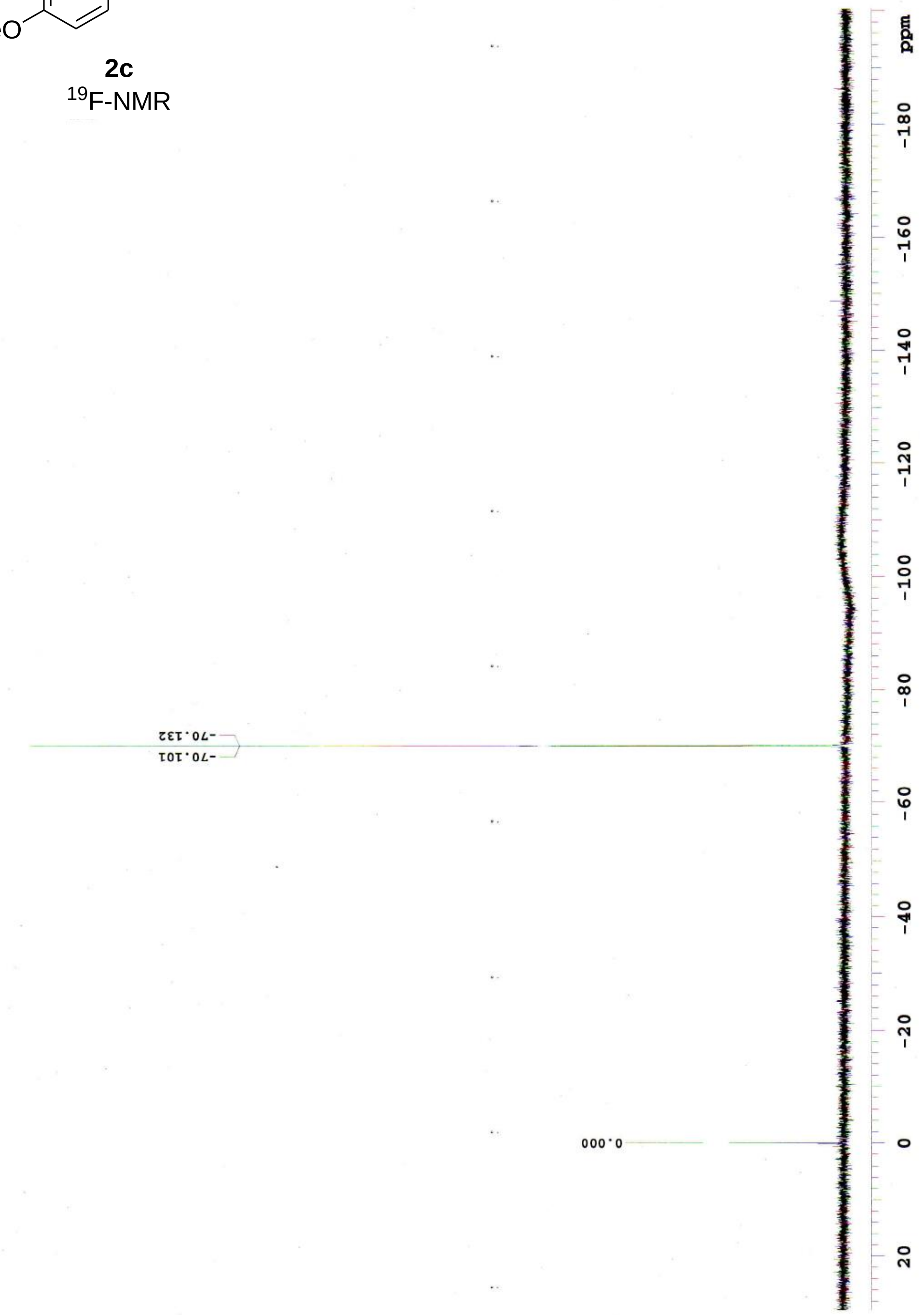
-70.132
-70.101
0.000
ppm
-180
-160
-140
-120
-100
-80
-60
-40
-20
0
20

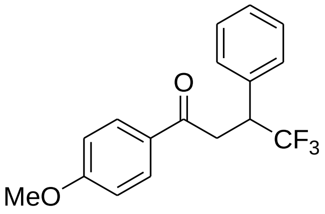
O
CF3
MeO

2c
^{13}C-NMR

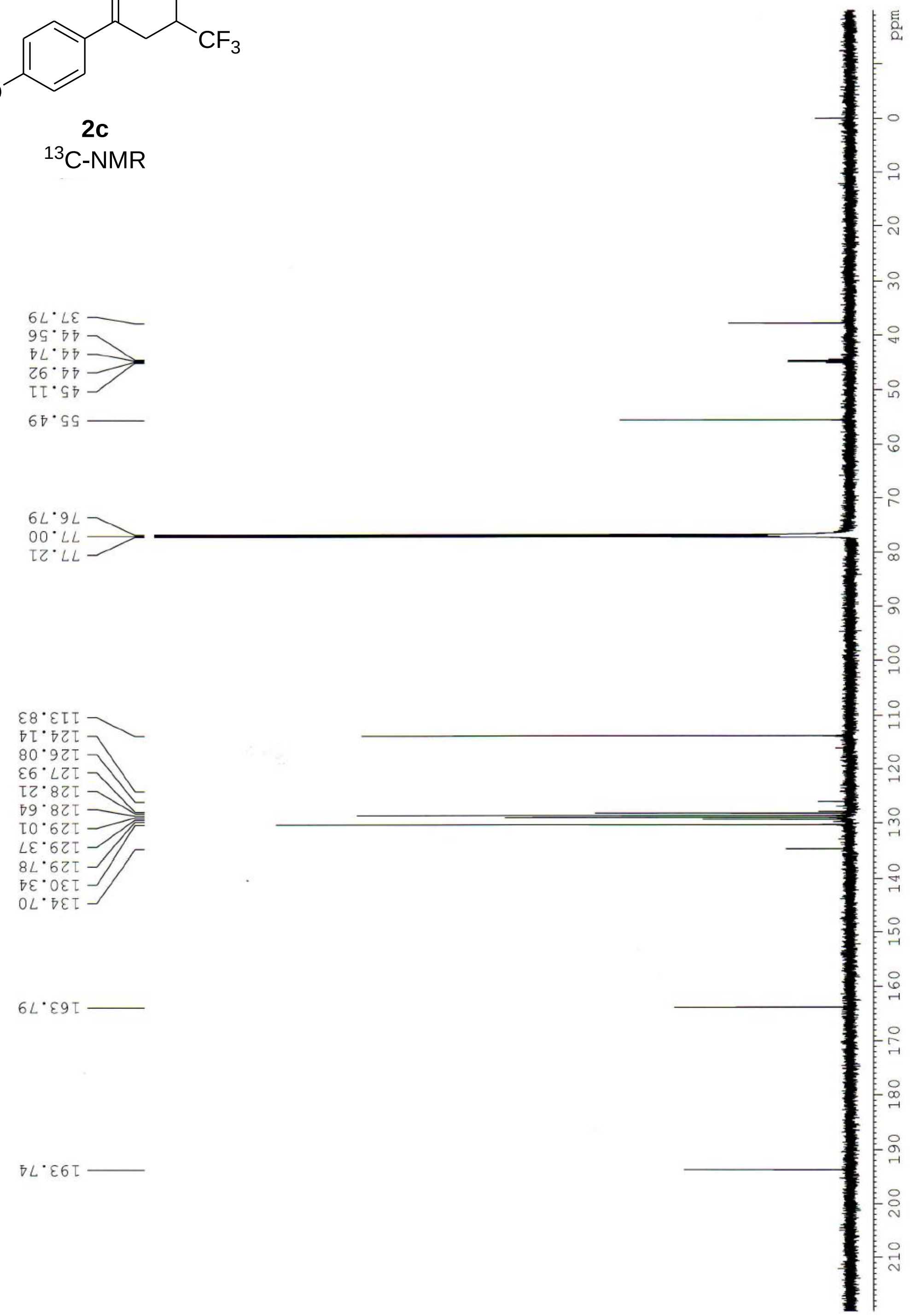
37.79
44.56
44.74
44.92
45.11
55.49
76.79
77.00
77.21
113.83
124.14
126.08
127.93
128.21
128.64
129.01
129.37
129.78
130.34
134.70
163.79
193.74
ppm
0
10
20
30
40
50
60
70
80
90
100
110
120
130
140
150
160
170
180
190
200
210

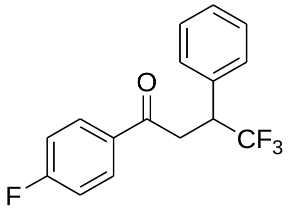
O
CF3
F

2d

^{1}H-NMR

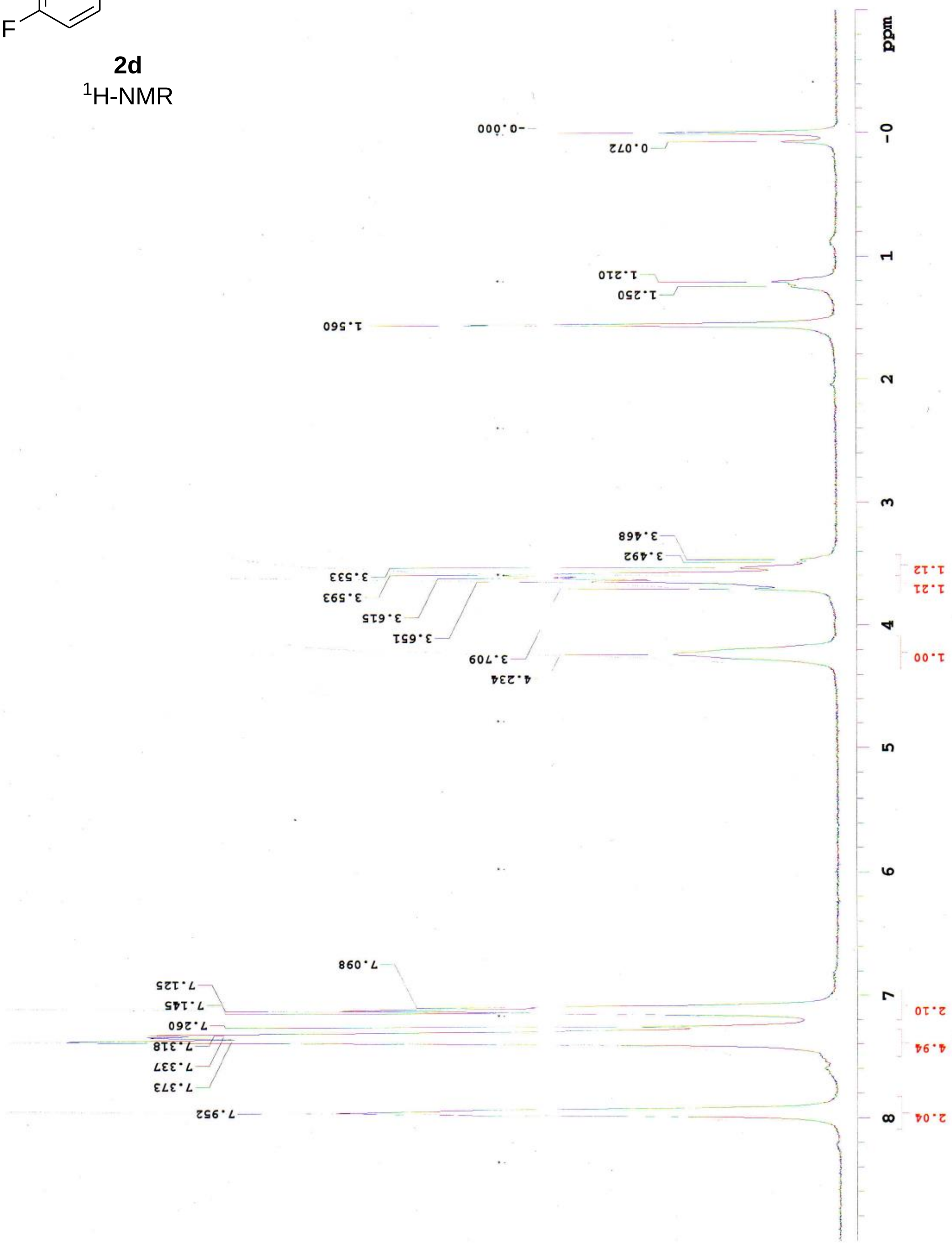
ppm
-0.000
0.072
1.210
1.250
1.560
3.468
3.492
3.533
3.593
3.615
3.651
3.709
4.234
7.098
7.125
7.145
7.260
7.318
7.337
7.373
7.952
1.12
1.21
1.00
2.10
4.94
2.04

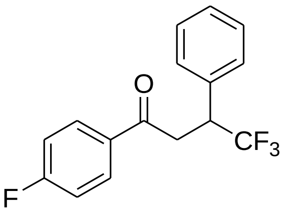

2d

^{19}F-NMR

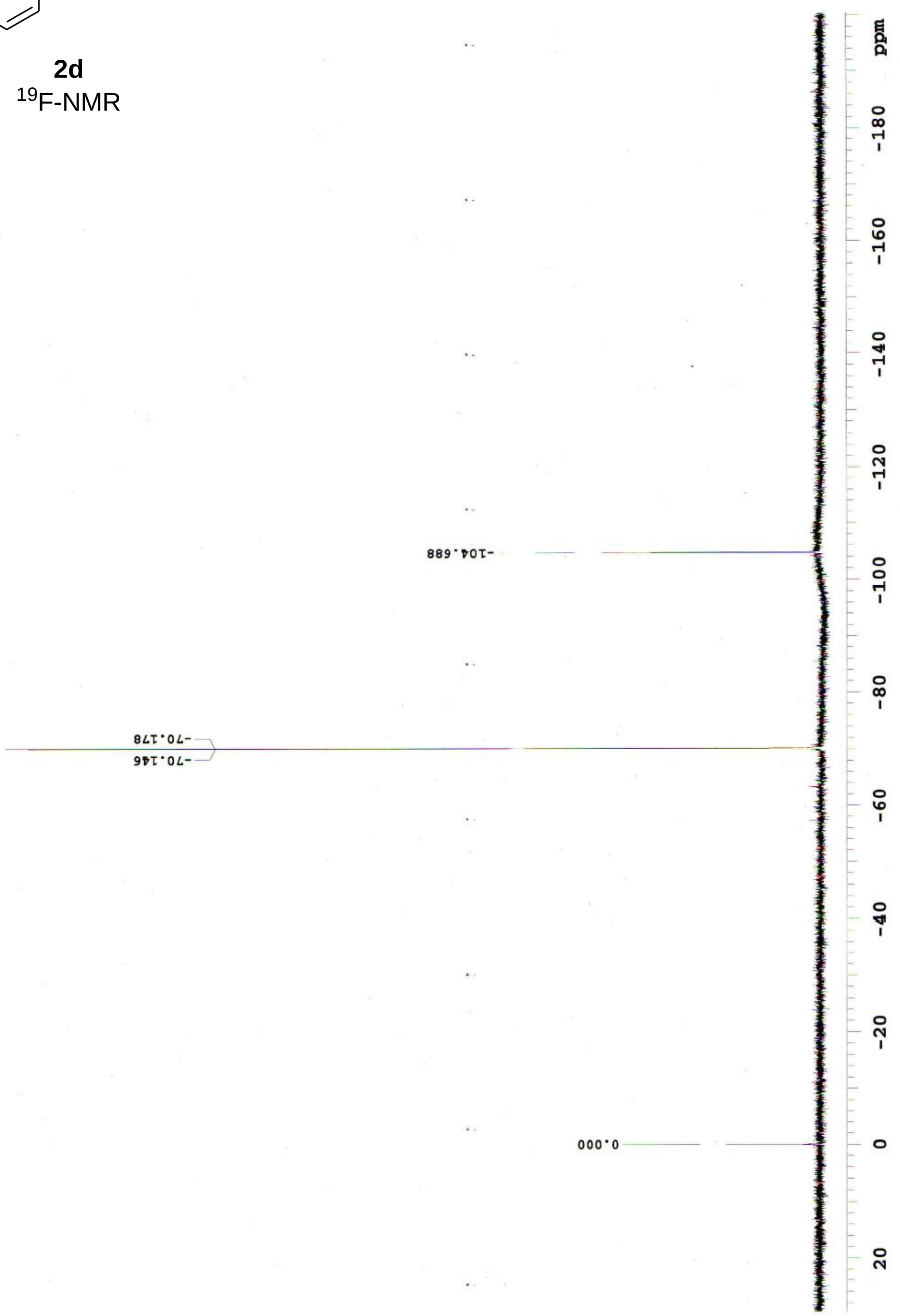

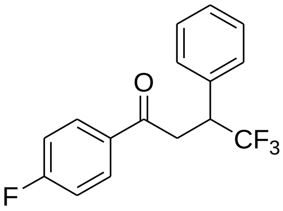
O
CF3
F

2d

^{13}C-NMR

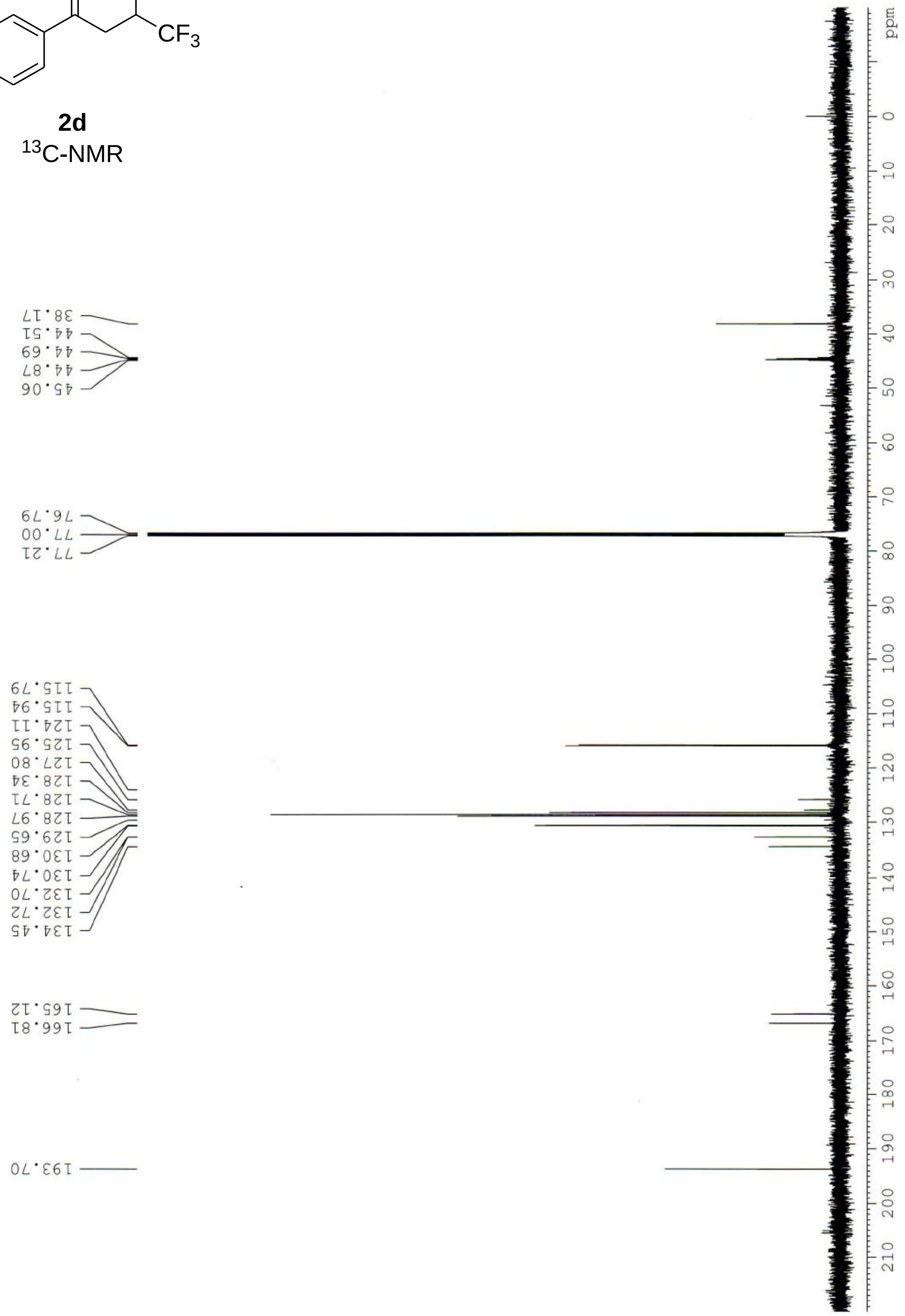
38.17
44.51
44.69
44.87
45.06
76.79
77.00
77.21
115.79
115.94
124.11
125.95
127.80
128.34
128.71
128.97
129.65
130.68
130.74
132.70
132.72
134.45
165.12
166.81
193.70
ppm

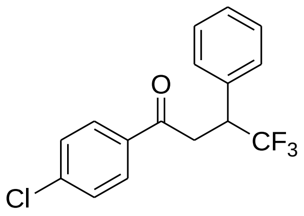

2e

^{1}H-NMR

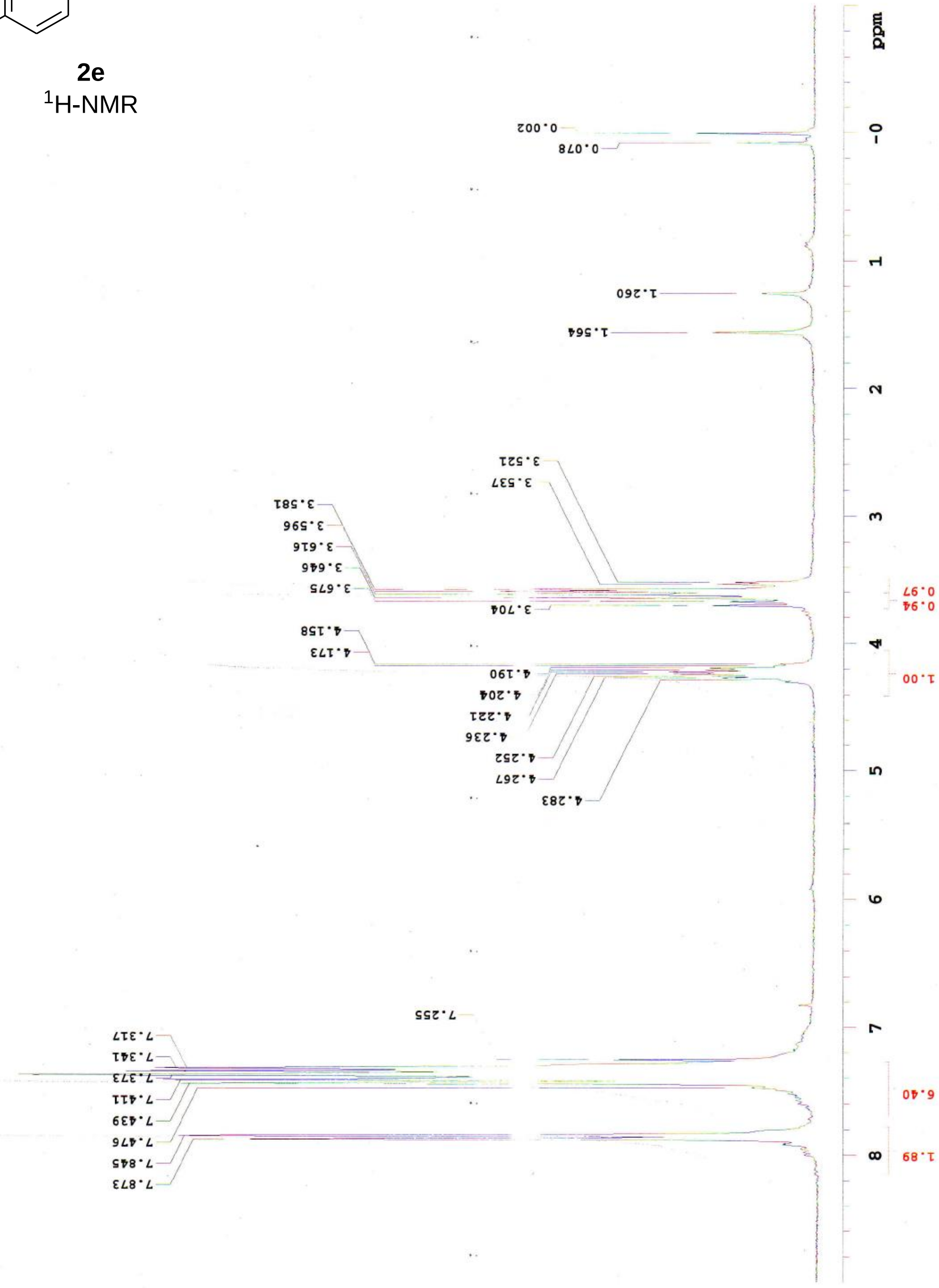
ppm
0.002
0.078
1.260
1.564
3.521
3.537
3.581
3.596
3.616
3.646
3.675
3.704
4.158
4.173
4.190
4.204
4.221
4.236
4.252
4.267
4.283
7.255
7.317
7.341
7.373
7.411
7.439
7.476
7.845
7.873
0.97
0.94
1.00
6.40
1.89

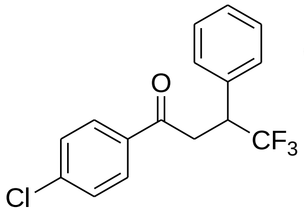

2e
^{19}F-NMR

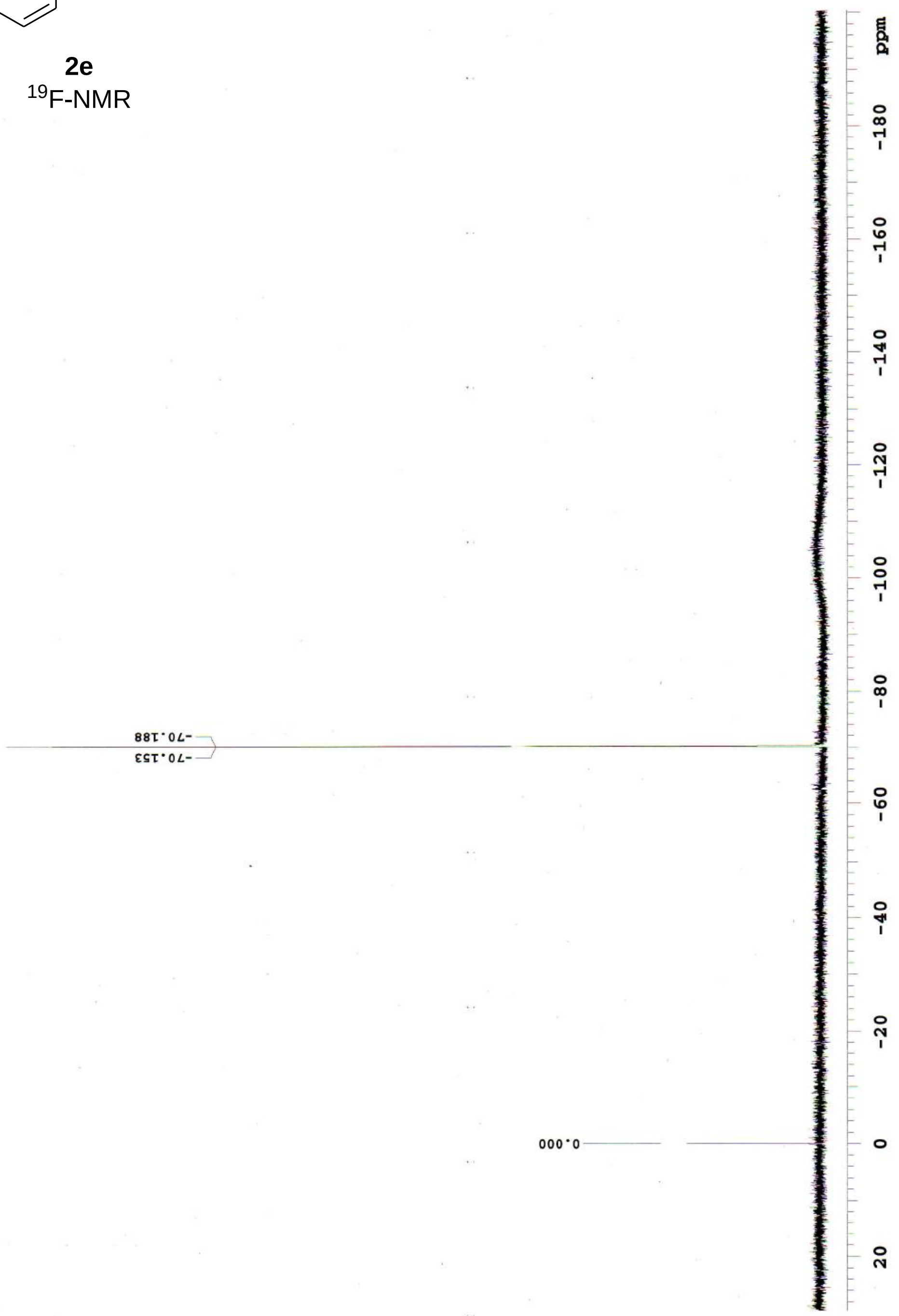

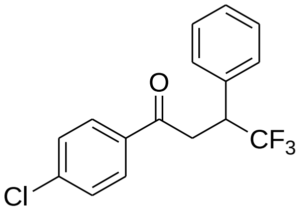
O
CF3
Cl

2e

^{13}C-NMR

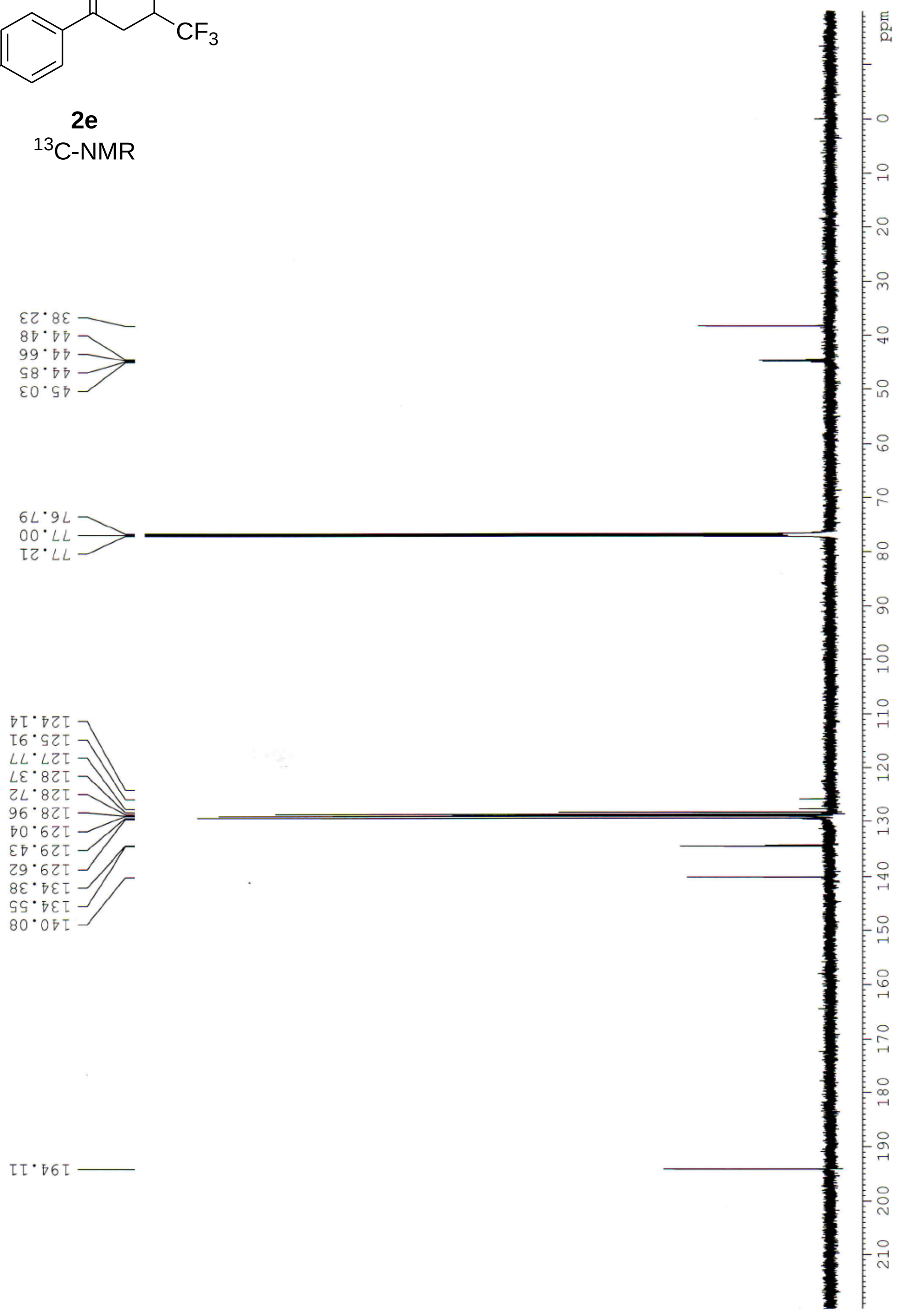
38.23
44.48
44.66
44.85
45.03
76.79
77.00
77.21
124.14
125.91
127.77
128.37
128.72
128.96
129.04
129.43
129.62
134.38
134.55
140.08
194.11
ppm

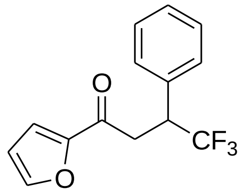
O
CF3
O

2f

^{1}H-NMR

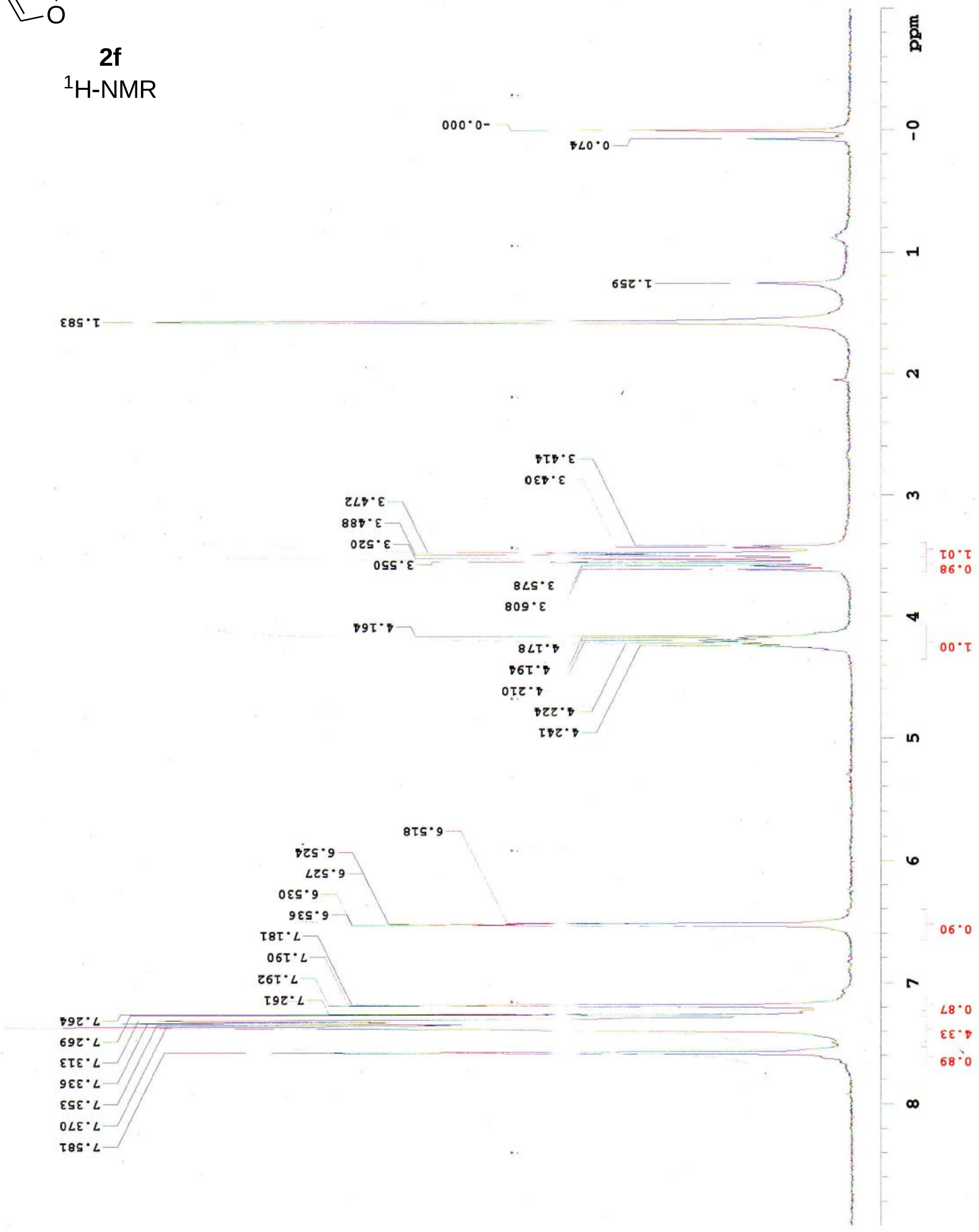
ppm
-0.000
0.074
1.259
1.583
3.414
3.430
3.472
3.488
3.520
3.550
3.578
3.608
4.164
4.178
4.194
4.210
4.224
4.241
6.518
6.524
6.527
6.530
6.536
7.181
7.190
7.192
7.261
7.264
7.269
7.313
7.336
7.353
7.370
7.581
1.01
0.98
1.00
0.90
0.87
4.33
0.89
-0
1
2
3
4
5
6
7
8

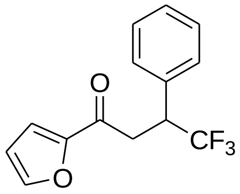

2f

^{19}F-NMR

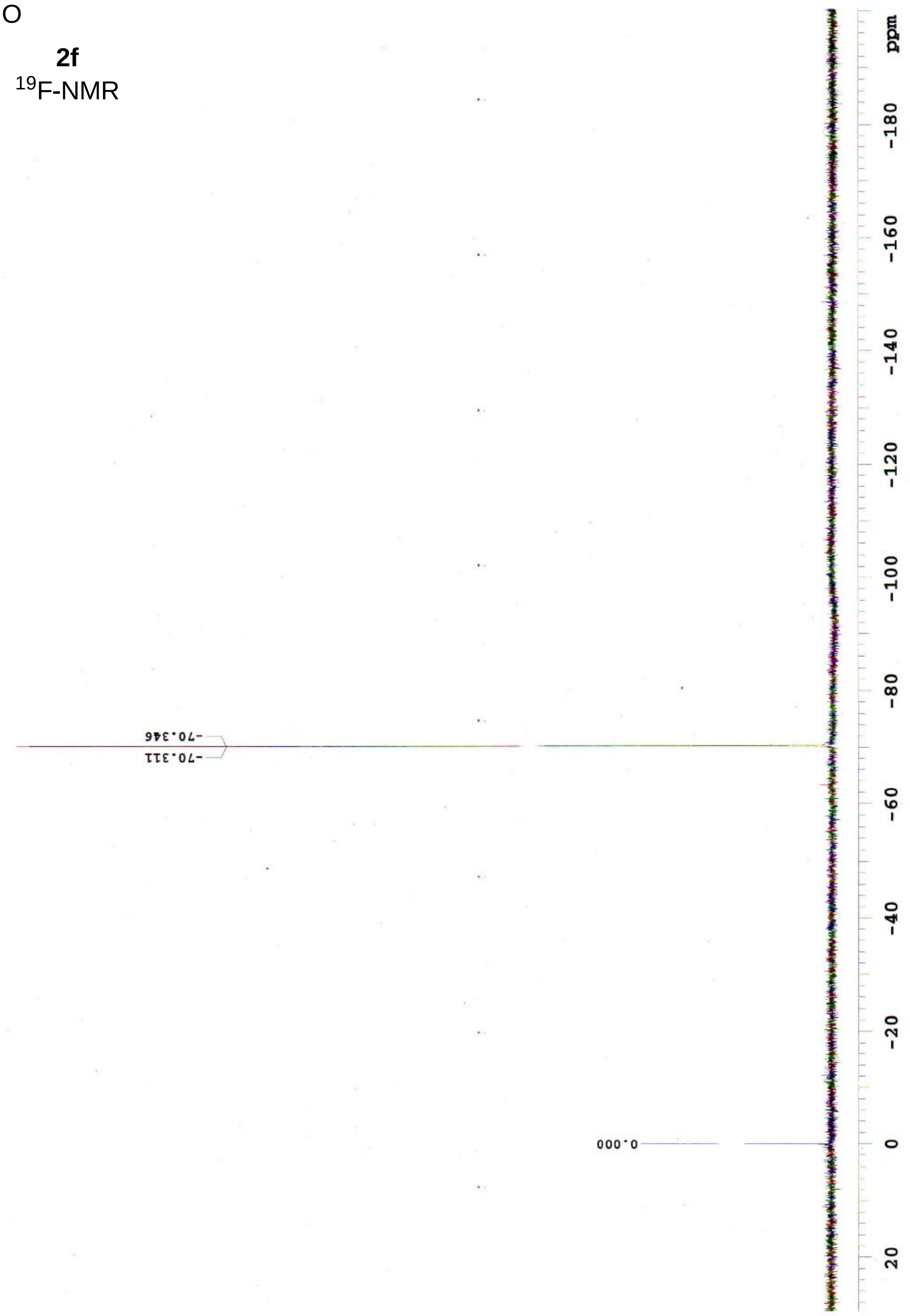

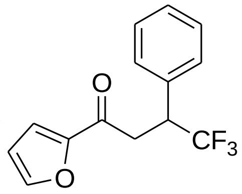

2f

^{13}C-NMR

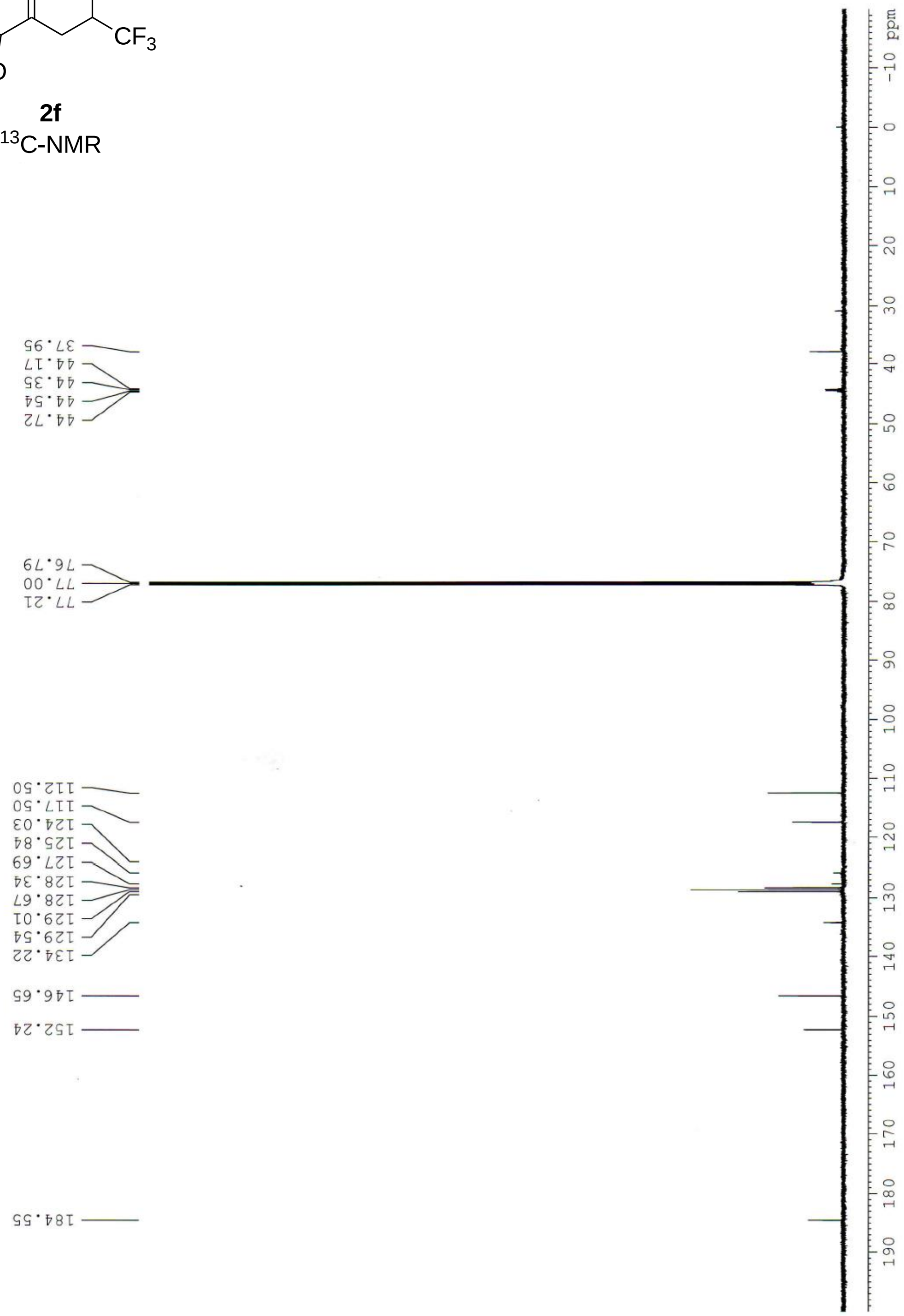
37.95
44.17
44.35
44.54
44.72
76.79
77.00
77.21
112.50
117.50
124.03
125.84
127.69
128.34
128.67
129.01
129.54
134.22
146.65
152.24
184.55
ppm

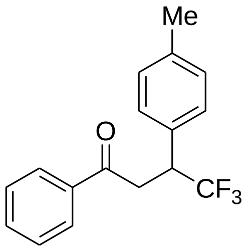

2g

^{1}H-NMR

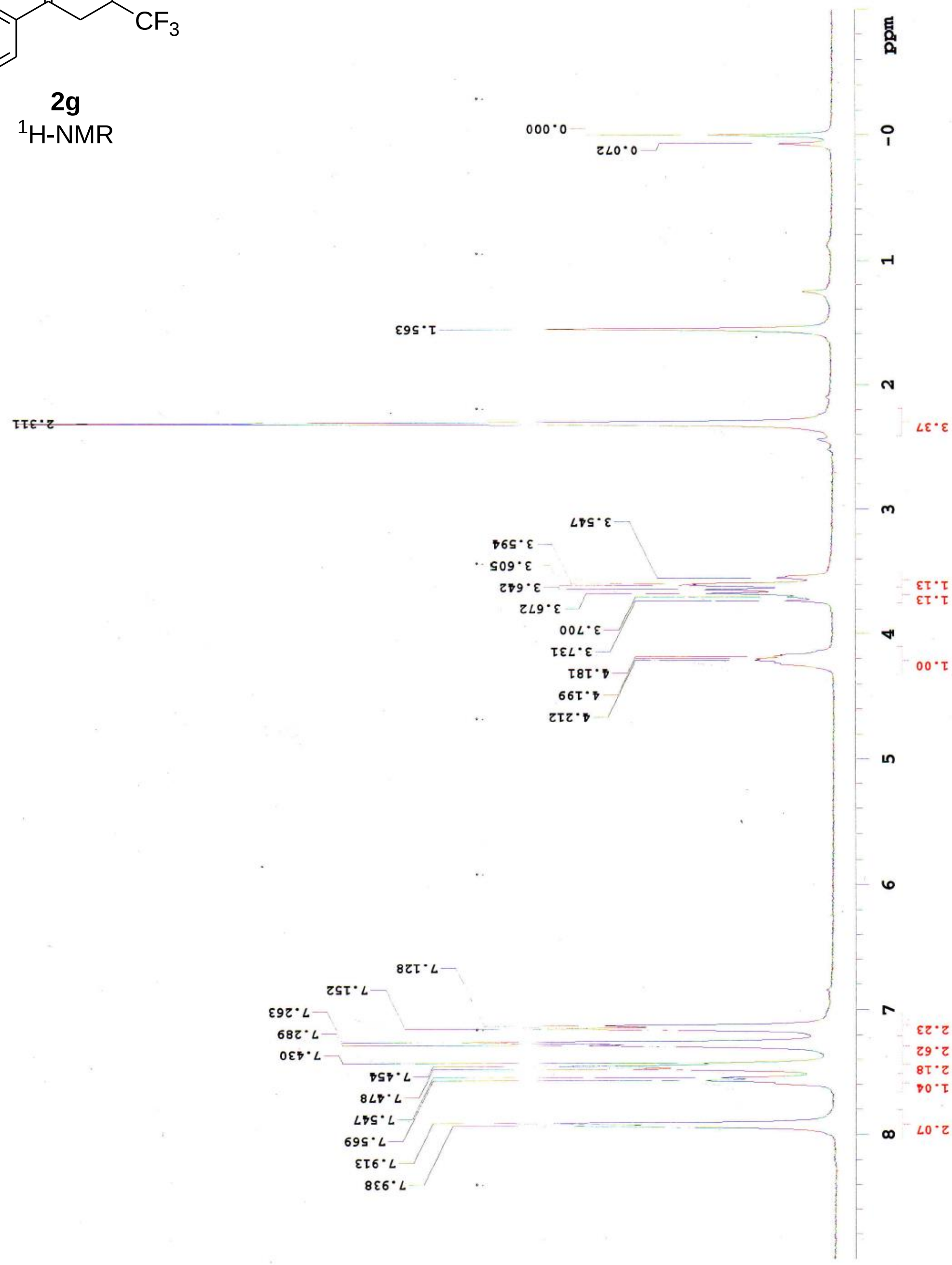

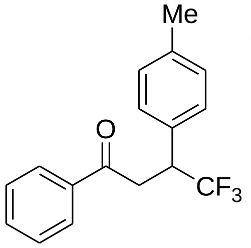

2g
^{19}F-NMR

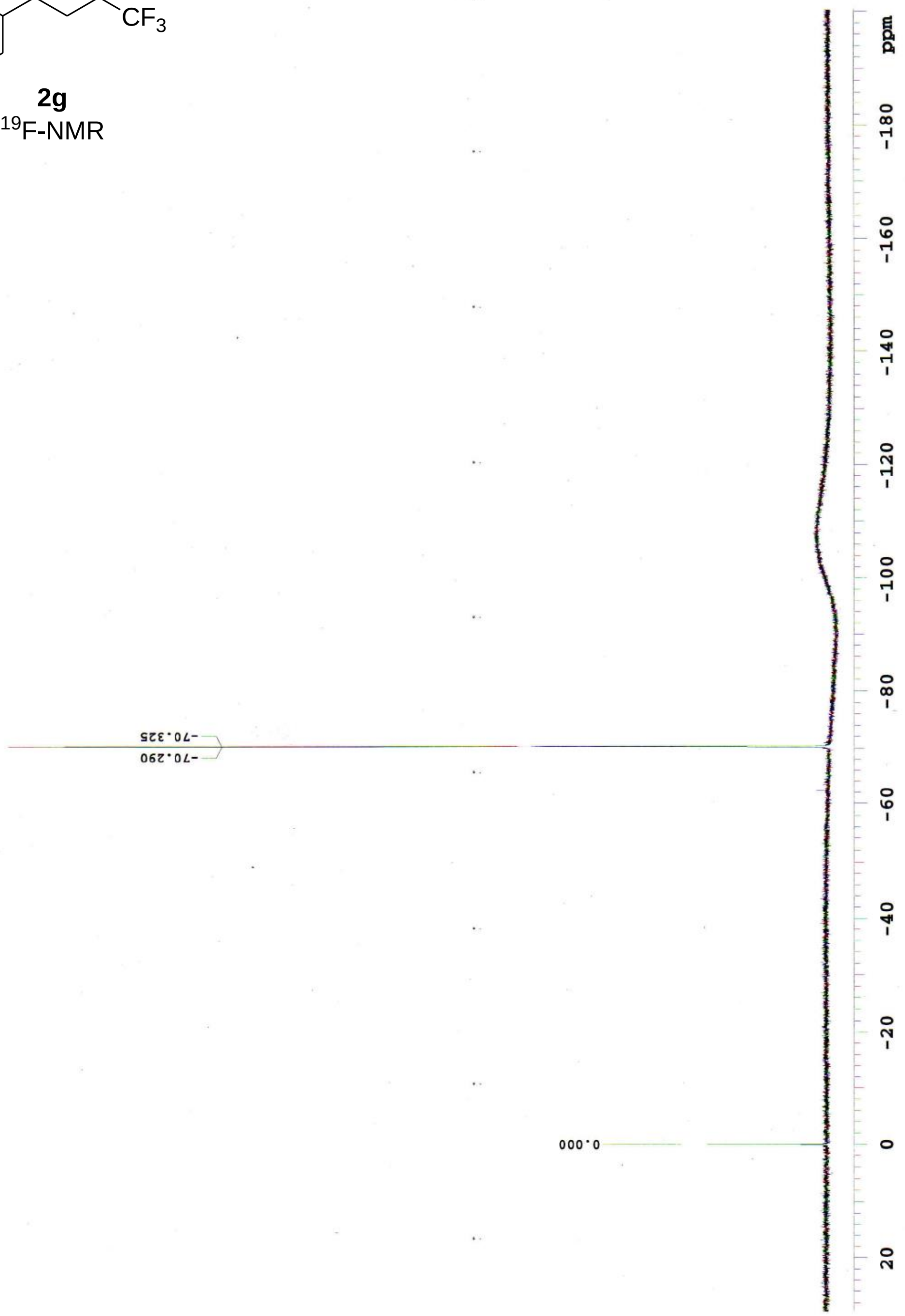

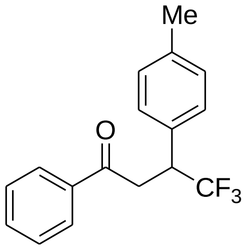

Me
O
CF3

2g

^{13}C-NMR

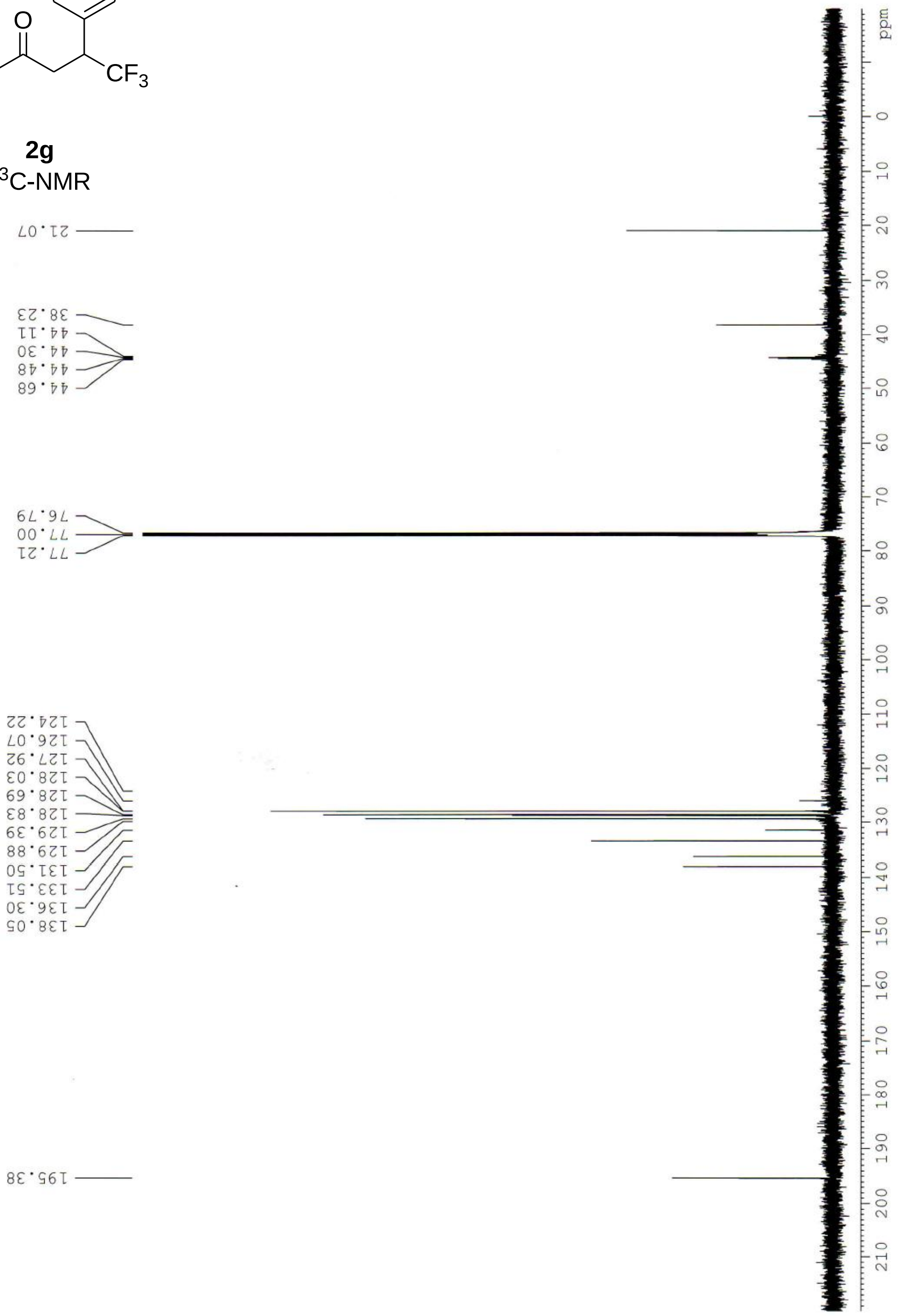

21.07
38.23
44.11
44.30
44.48
44.68
76.79
77.00
77.21
124.22
126.07
127.92
128.03
128.69
128.83
129.39
129.88
131.50
133.51
136.30
138.05
195.38
ppm
0
10
20
30
40
50
60
70
80
90
100
110
120
130
140
150
160
170
180
190
200
210

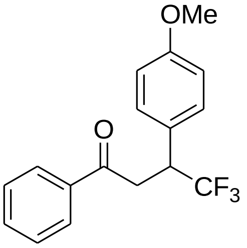

2h

^{1}H-NMR

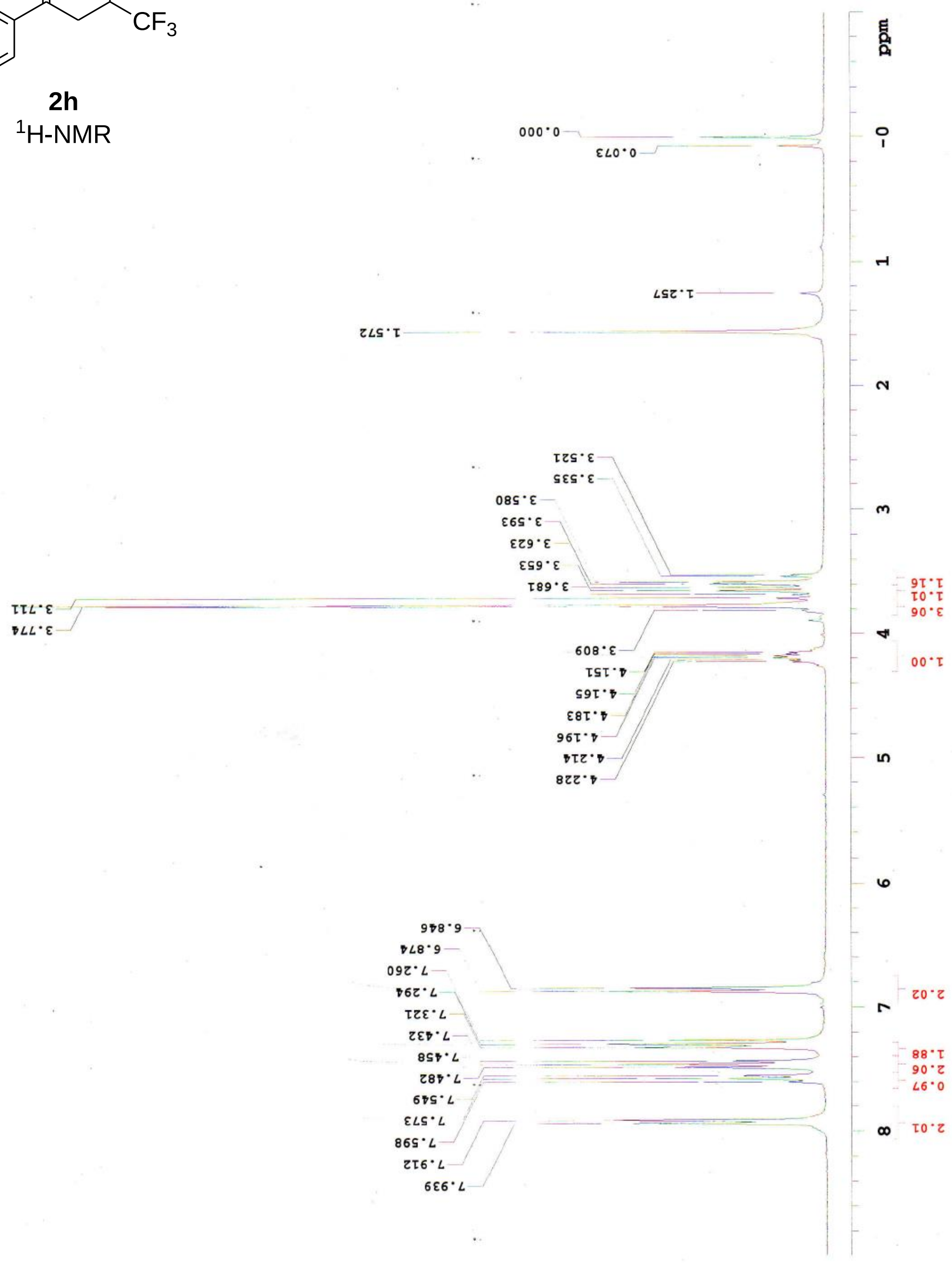

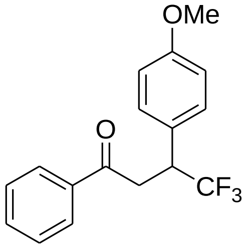
OMe
O
CF3

2h

^{19}F-NMR

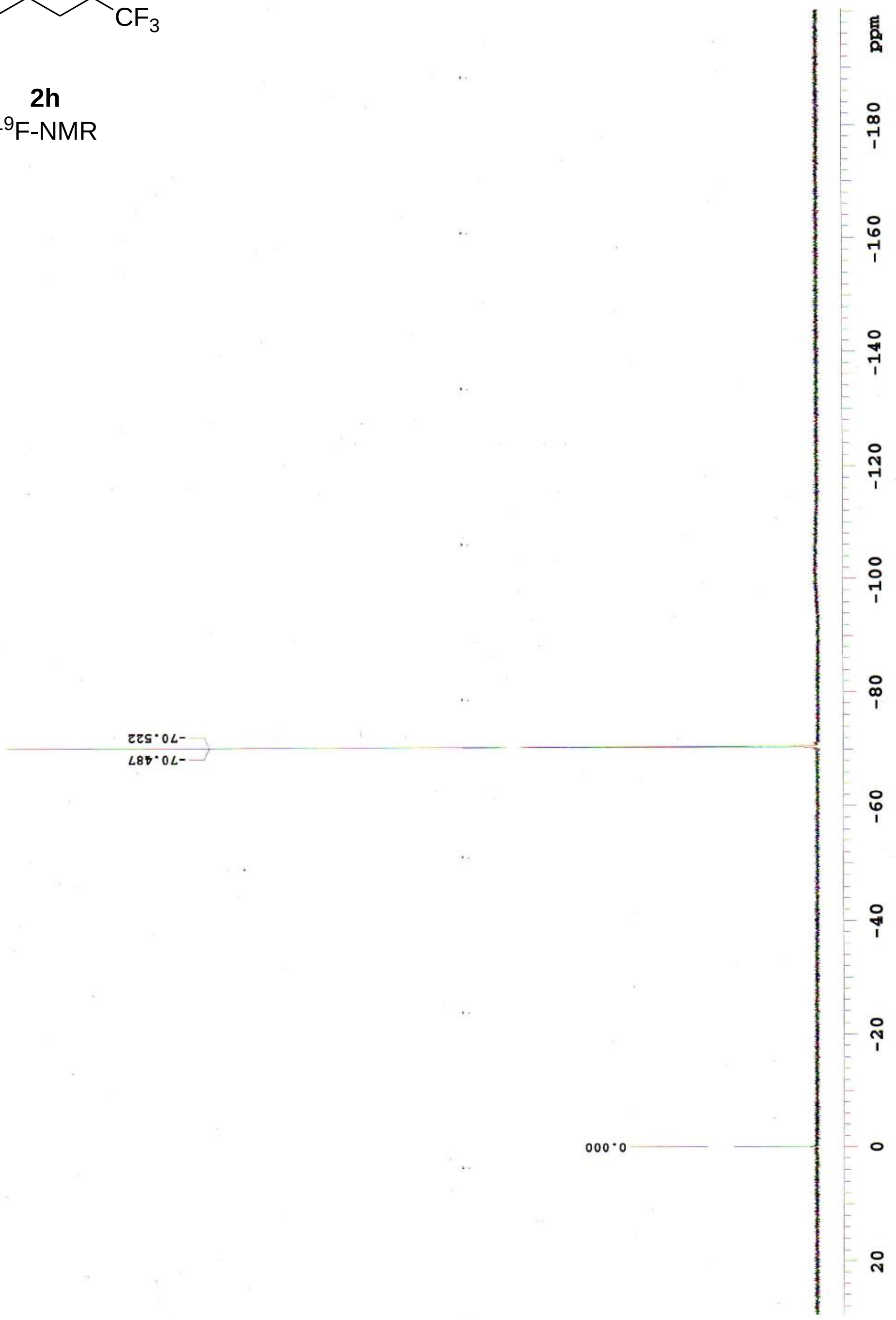
-70.522
-70.487
0.000
ppm
-180
-160
-140
-120
-100
-80
-60
-40
-20
0
20

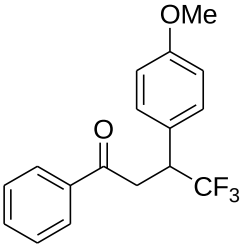
OMe
O
CF3

2h

^{13}C-NMR

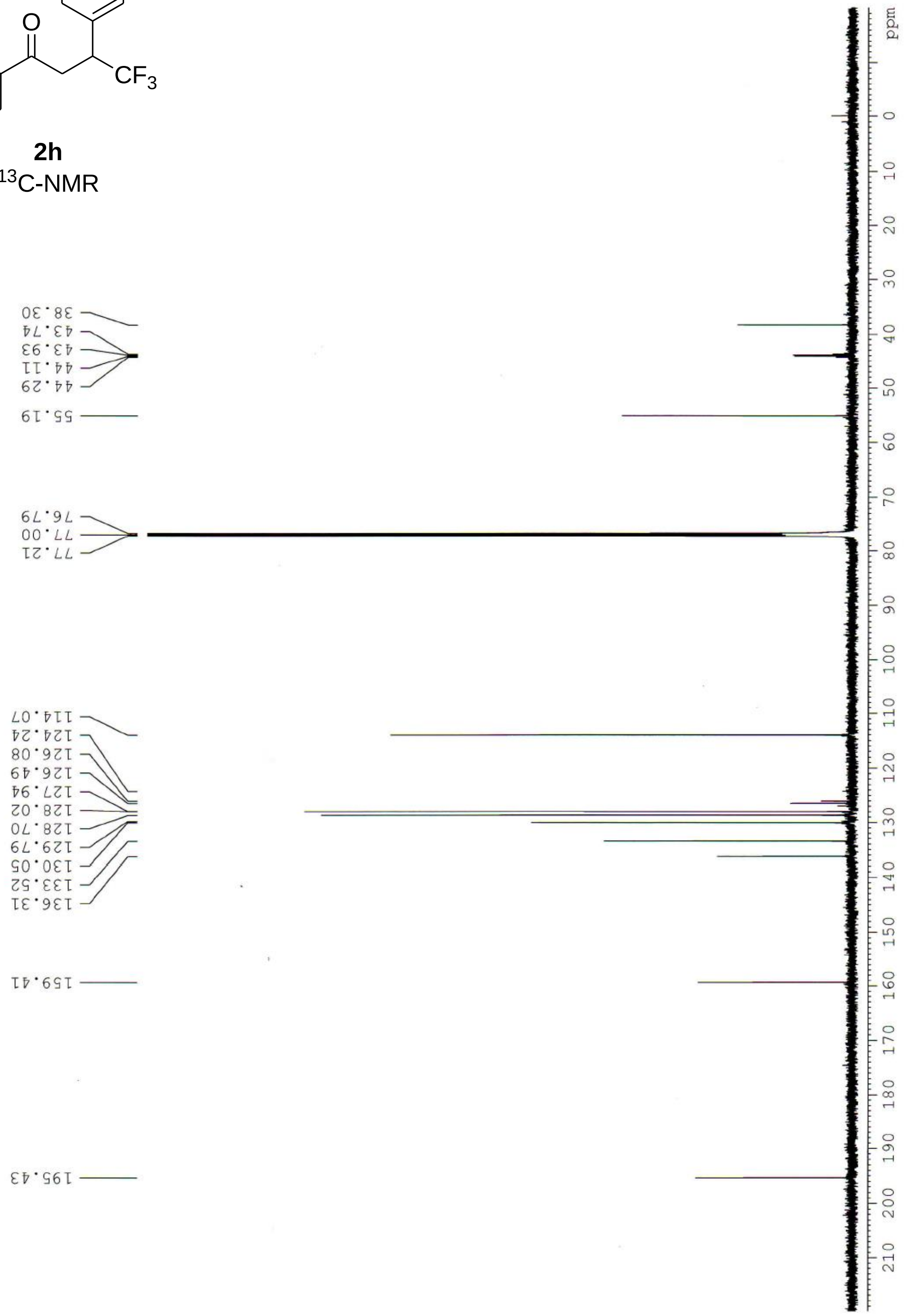
38.30
43.74
43.93
44.11
44.29
55.19
76.79
77.00
77.21
114.07
124.24
126.08
126.49
127.94
128.02
128.70
129.79
130.05
133.52
136.31
159.41
195.43
ppm
0
10
20
30
40
50
60
70
80
90
100
110
120
130
140
150
160
170
180
190
200
210

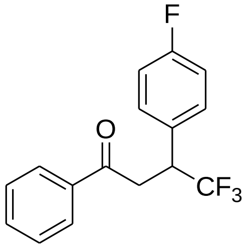

2i

^{1}H-NMR

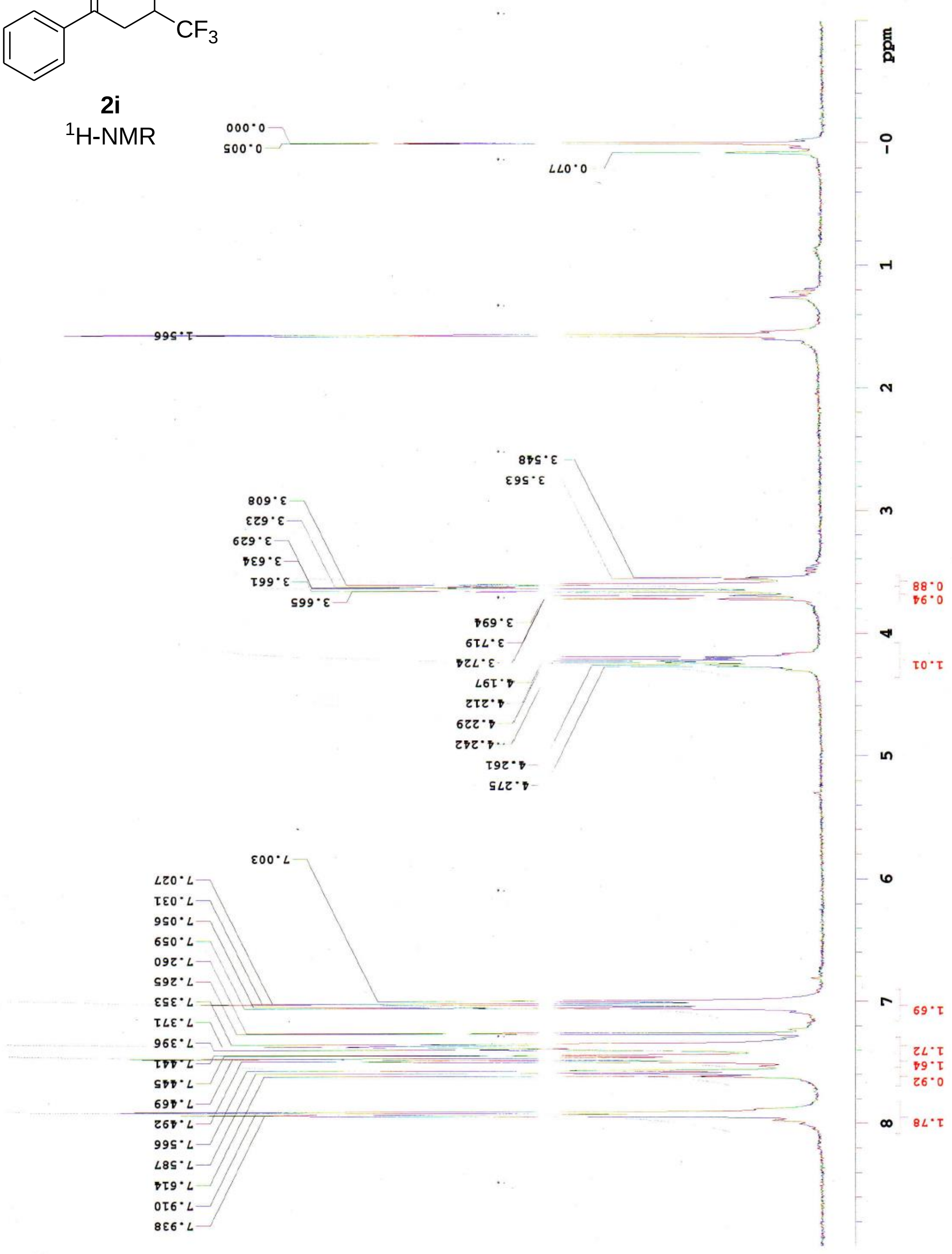

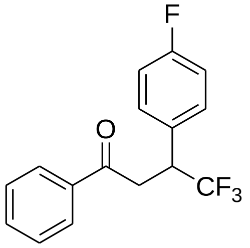
F
O
CF3

2i

^{13}C-NMR

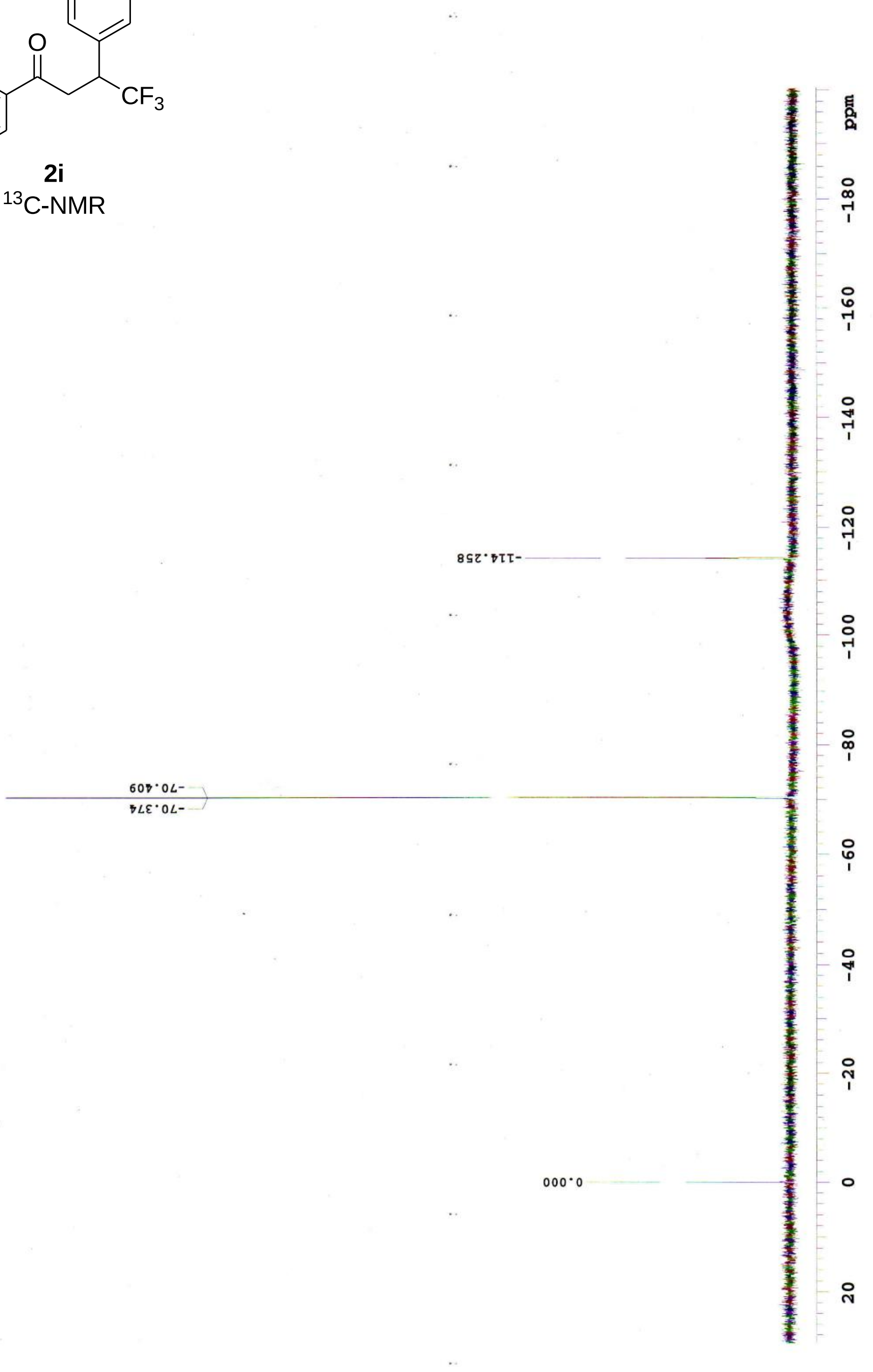
-114.258
-70.409
-70.374
0.000
ppm
-180
-160
-140
-120
-100
-80
-60
-40
-20
0
20

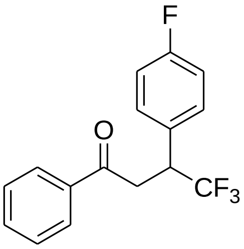
F
O
CF3

2i

^{13}C-NMR

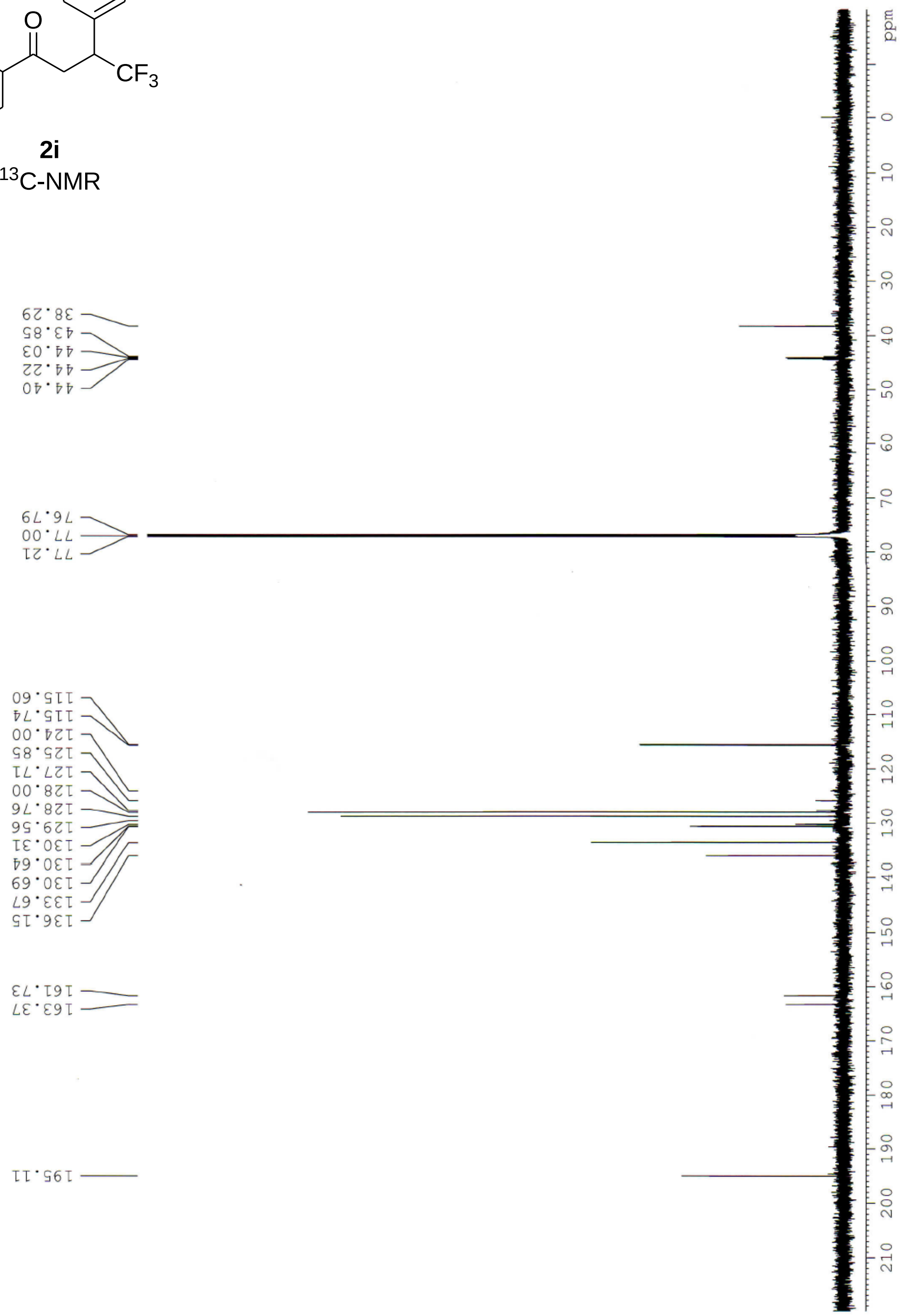
38.29
43.85
44.03
44.22
44.40
76.79
77.00
77.21
115.60
115.74
124.00
125.85
127.71
128.00
128.76
129.56
130.31
130.64
130.69
133.67
136.15
161.73
163.37
195.11
ppm

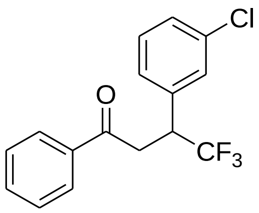

2j

^{1}H-NMR

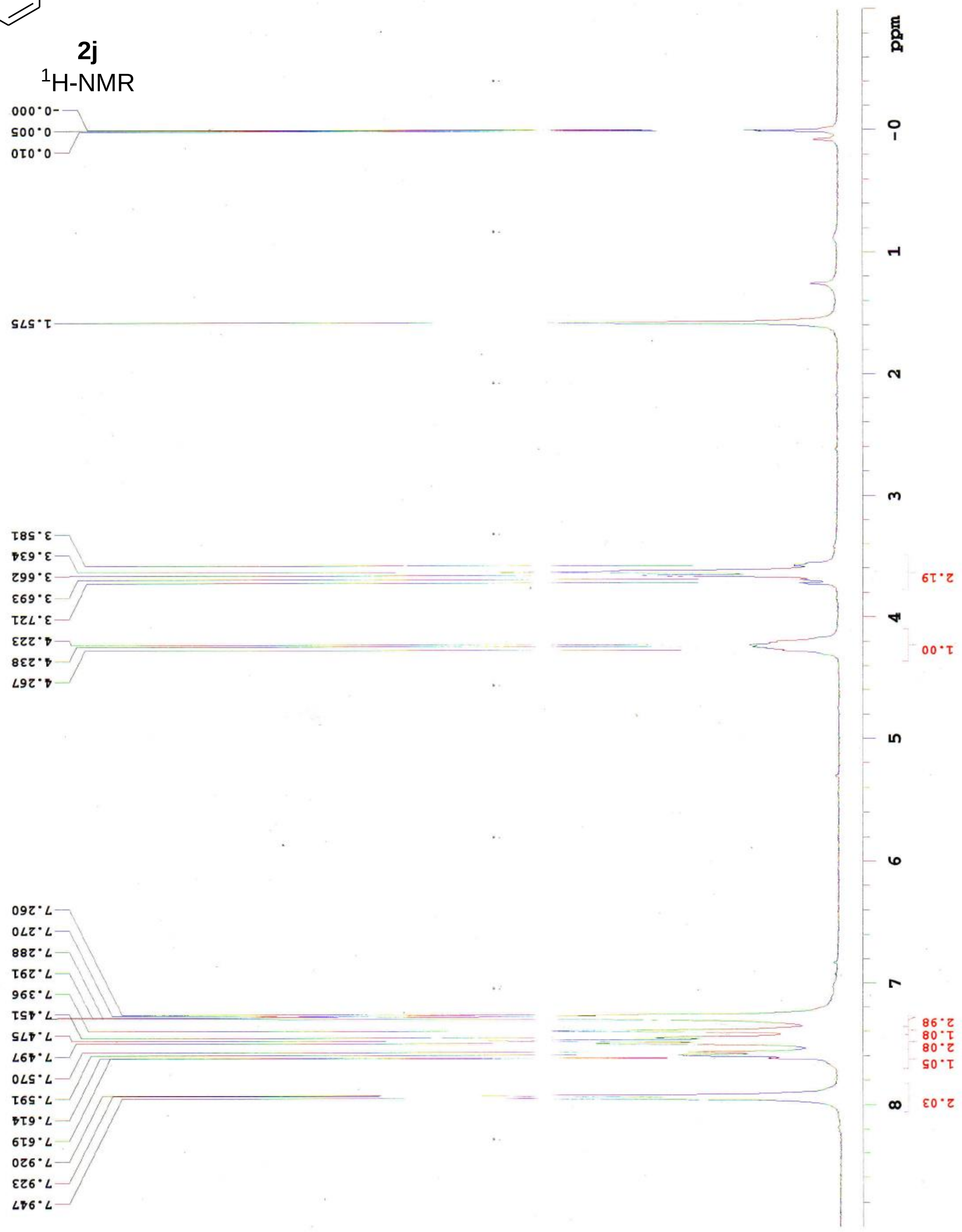

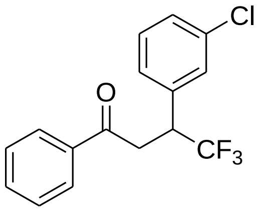

2j

^{19}F-NMR

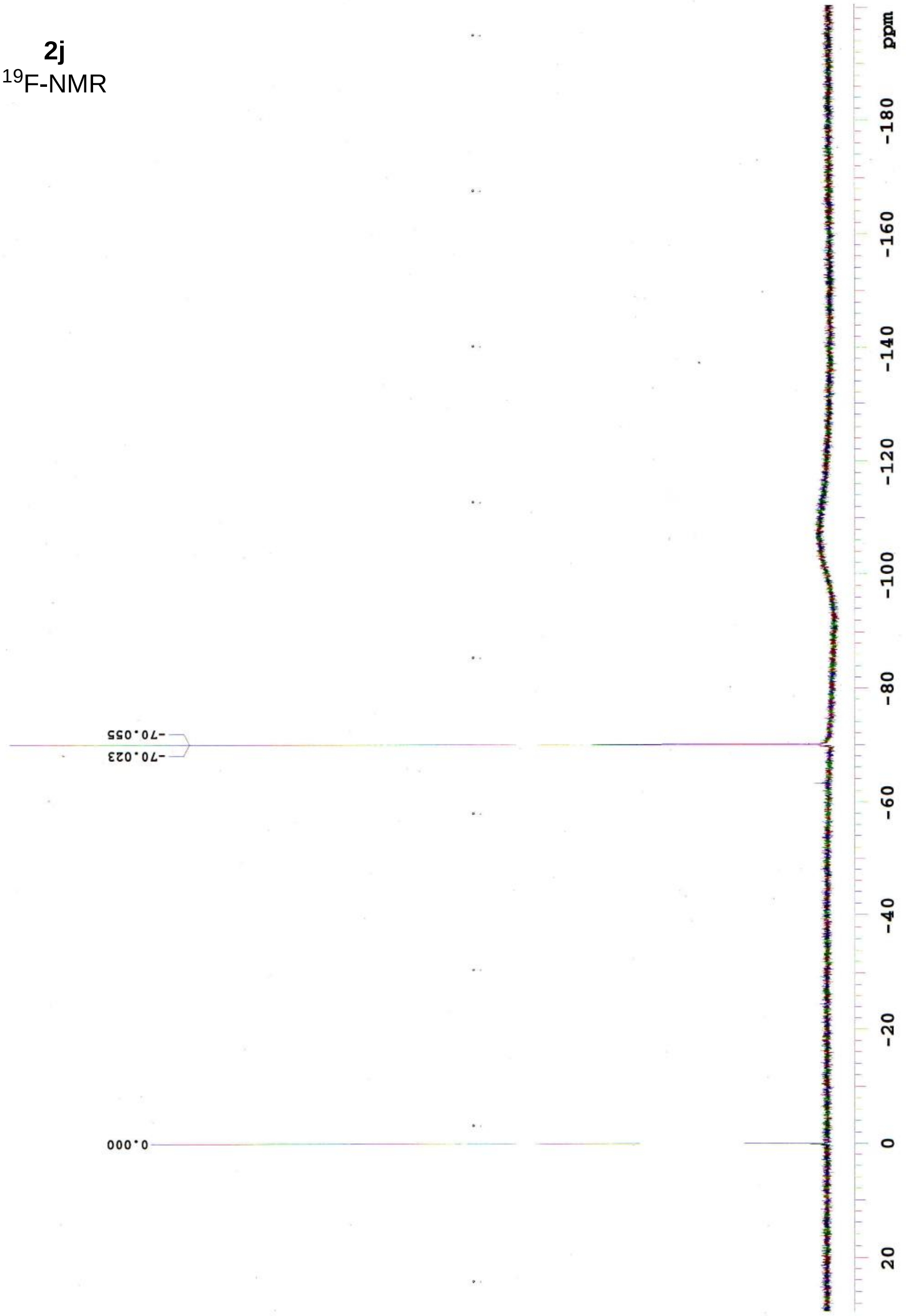

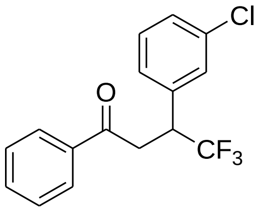
Cl
O
CF3

2j
^{13}C-NMR

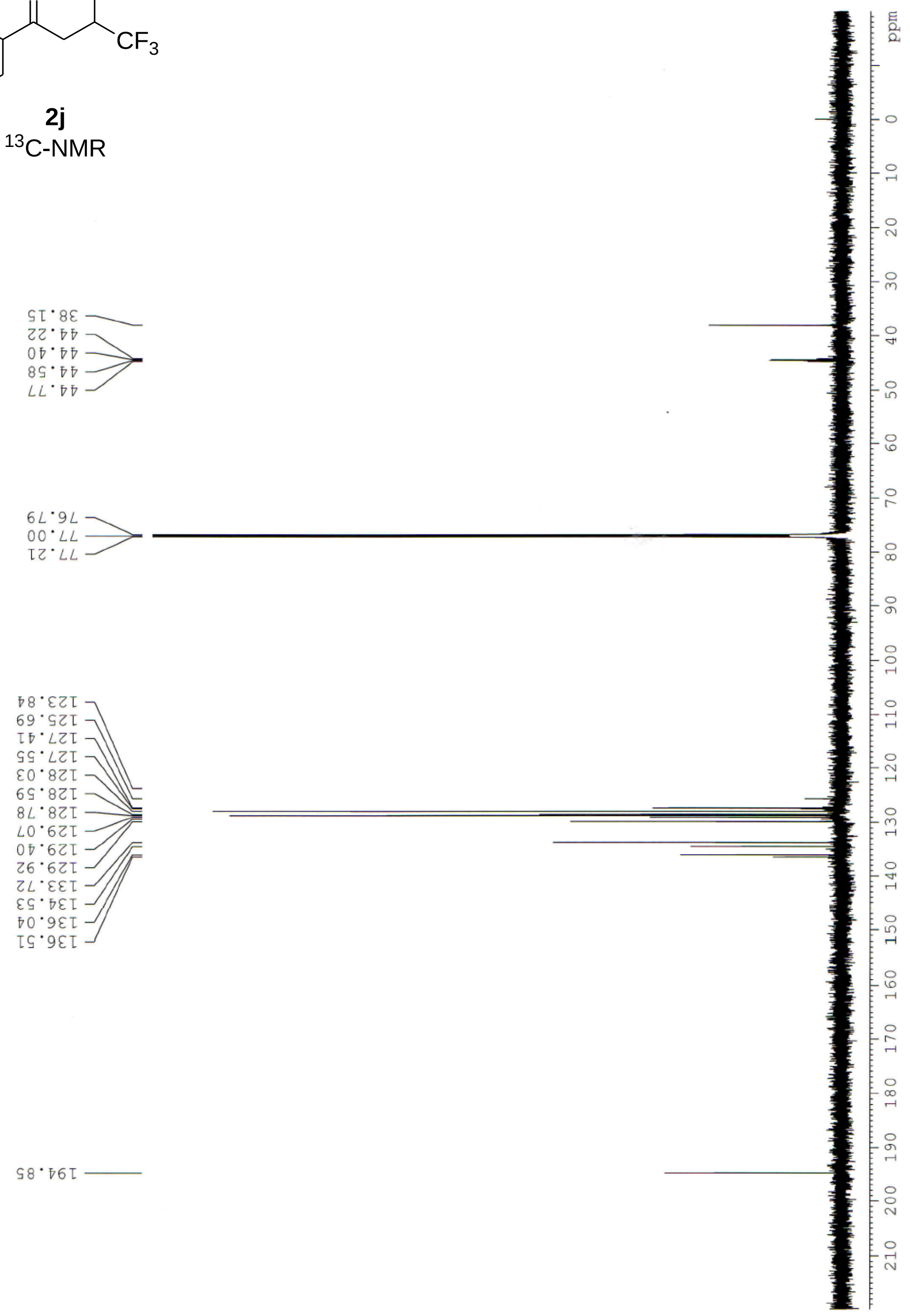
38.15
44.22
44.40
44.58
44.77
76.79
77.00
77.21
123.84
125.69
127.41
127.55
128.03
128.59
128.78
129.07
129.40
129.92
133.72
134.53
136.04
136.51
194.85
ppm

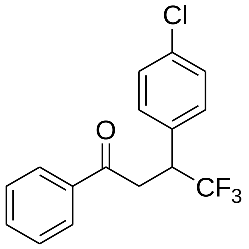
Cl
O
CF3

2k

^{1}H-NMR

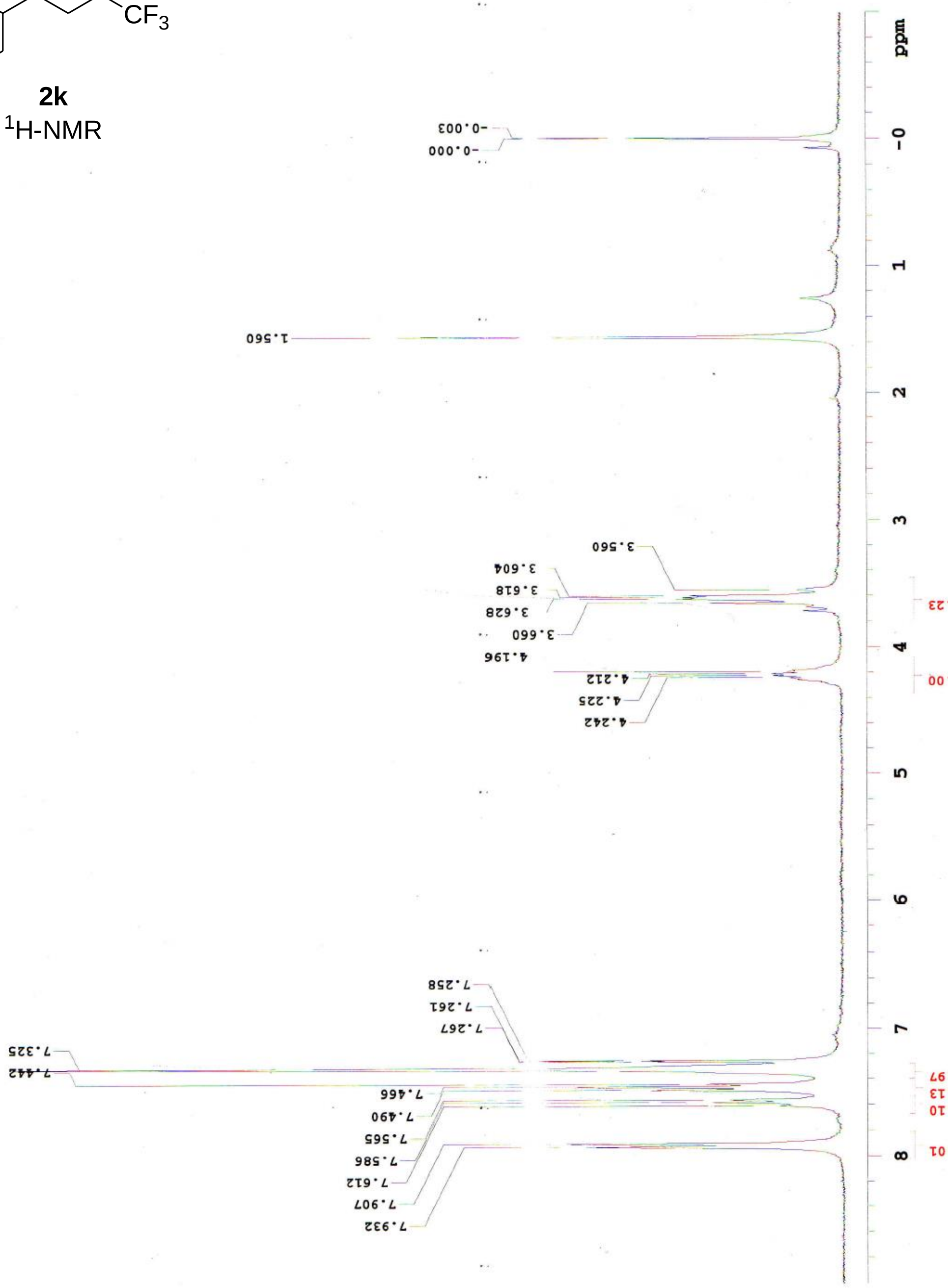
-0.003
-0.000
1.560
3.560
3.604
3.618
3.628
3.660
4.196
4.212
4.225
4.242
7.258
7.261
7.267
7.325
7.442
7.466
7.490
7.565
7.586
7.612
7.907
7.932
2.23
1.00
3.97
2.13
1.10
2.01
ppm

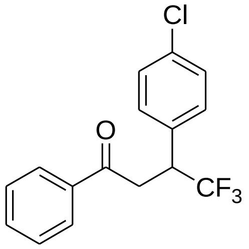

2k
^{19}F-NMR

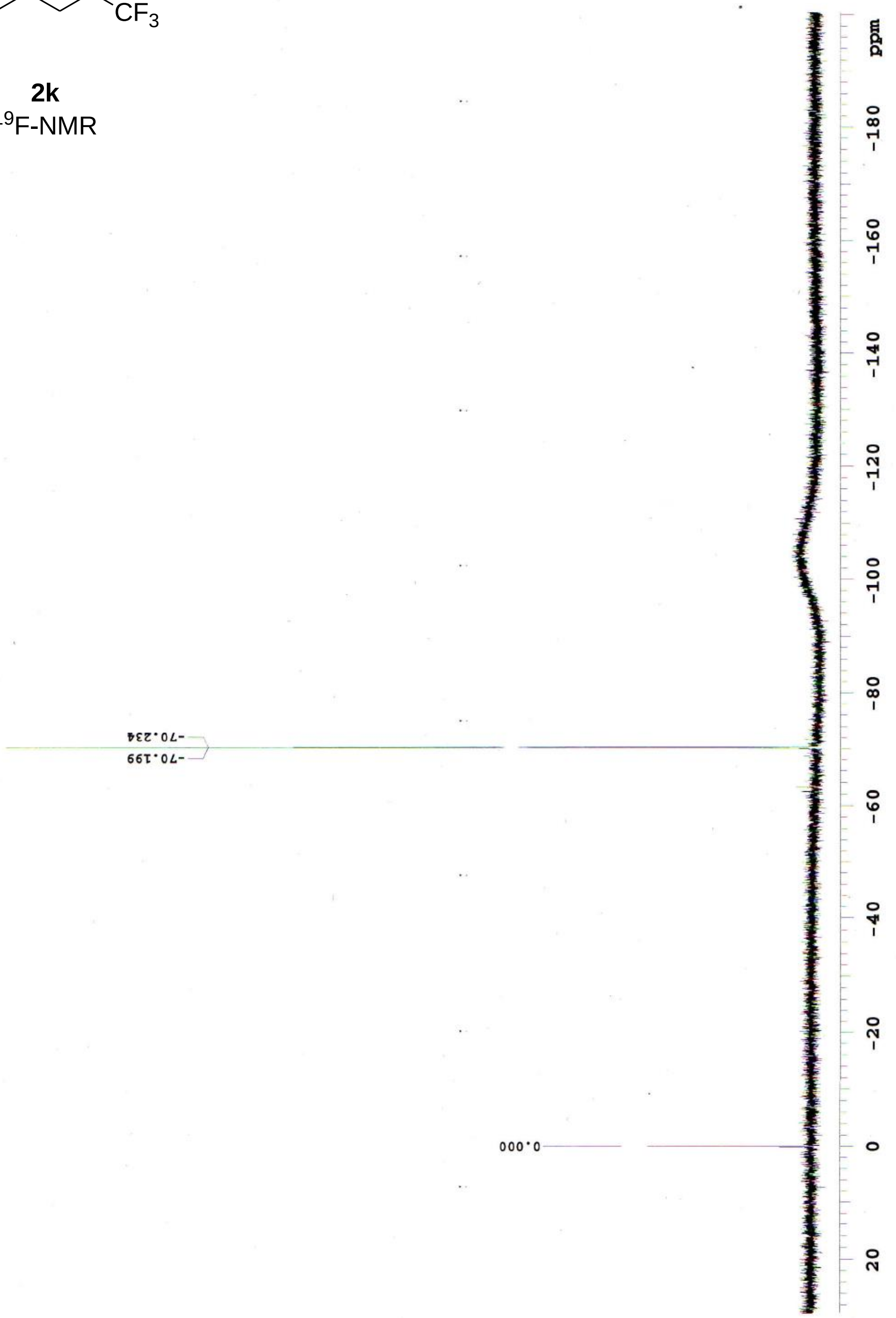

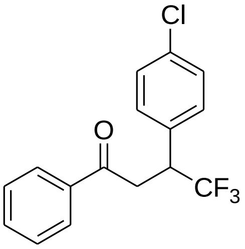
Cl
O
CF3

2k
^{13}C-NMR

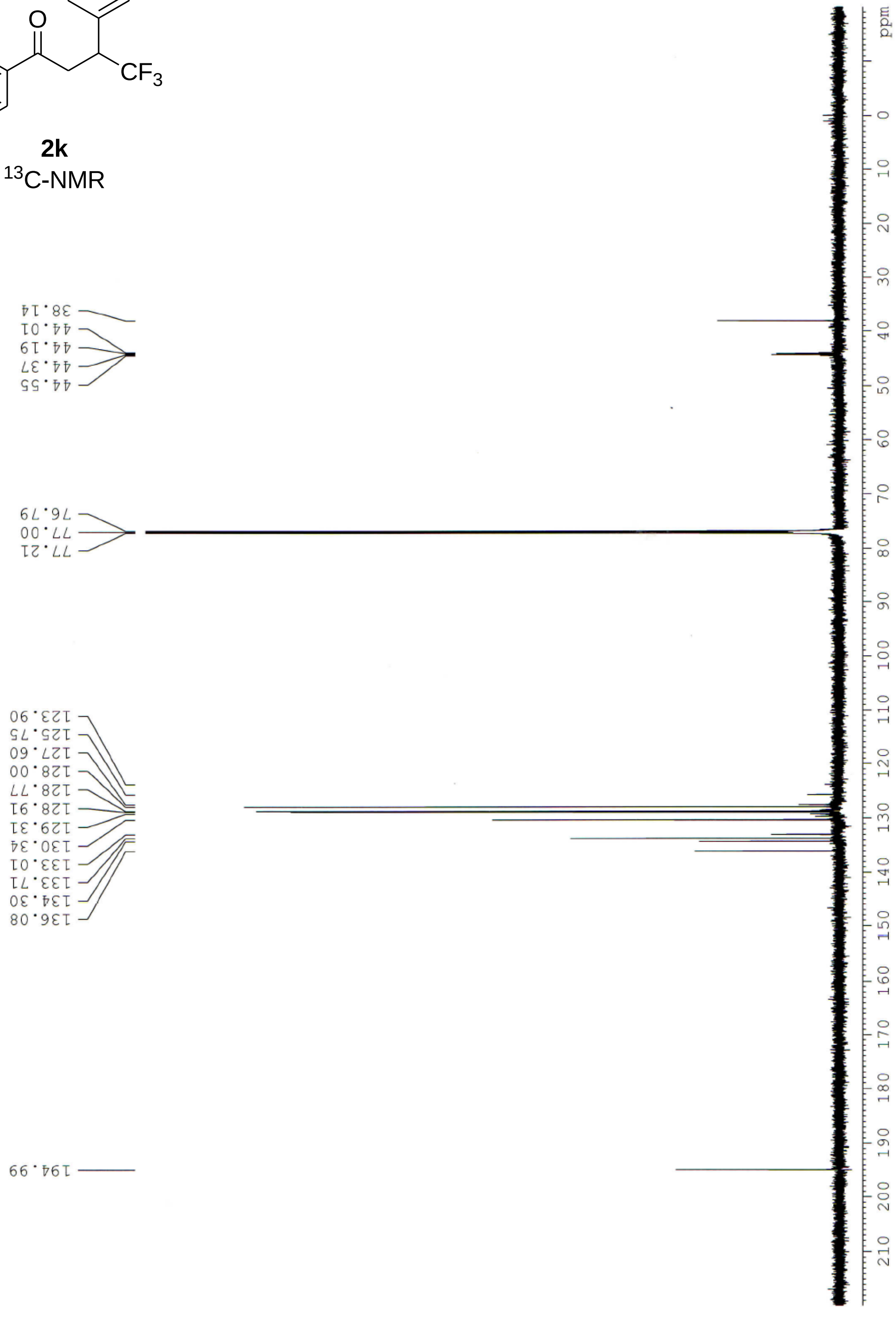
38.14
44.01
44.19
44.37
44.55
76.79
77.00
77.21
123.90
125.75
127.60
128.00
128.77
128.91
129.31
130.34
133.01
133.71
134.30
136.08
194.99
ppm

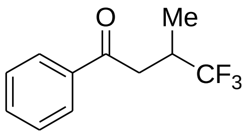
O
Me
CF3

2l

^{1}H-NMR

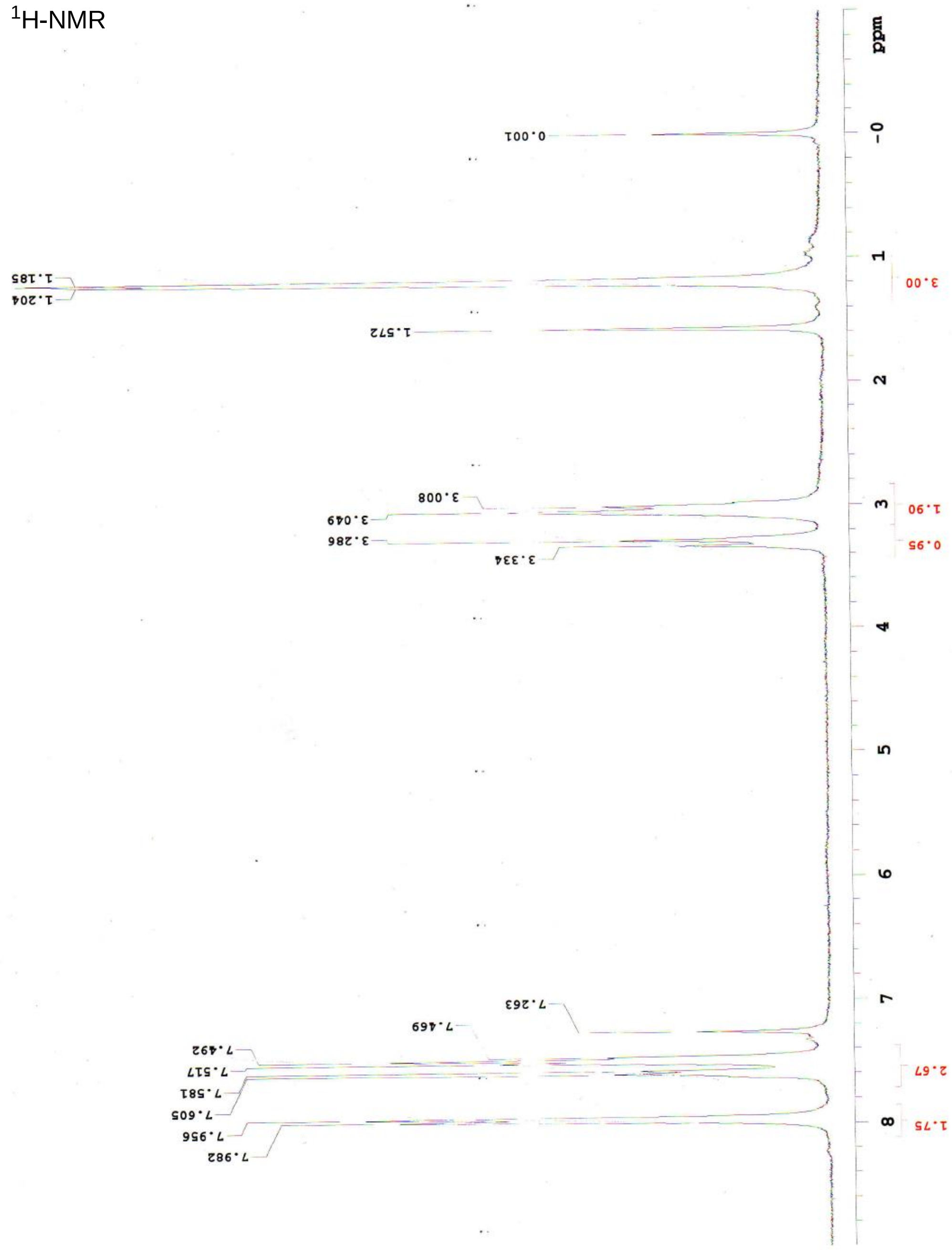
0.001
1.185
1.204
1.572
3.008
3.049
3.286
3.334
7.263
7.469
7.492
7.517
7.581
7.605
7.956
7.982
3.00
1.90
0.95
2.67
1.75
ppm

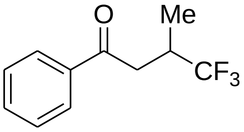

2l

^{19}F-NMR

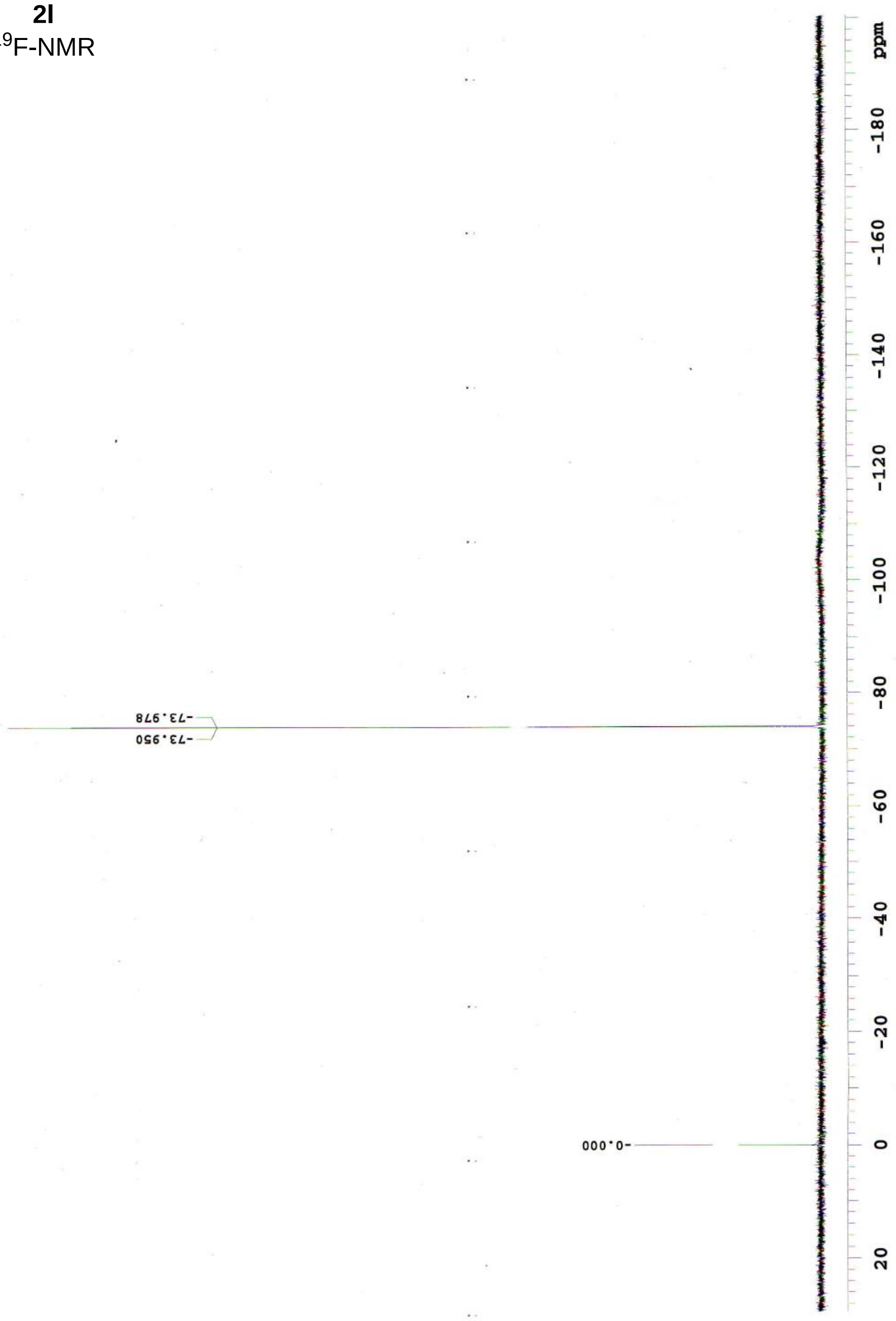

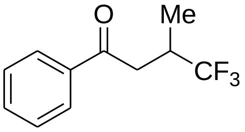
O
Me
CF3

2l

^{13}C-NMR

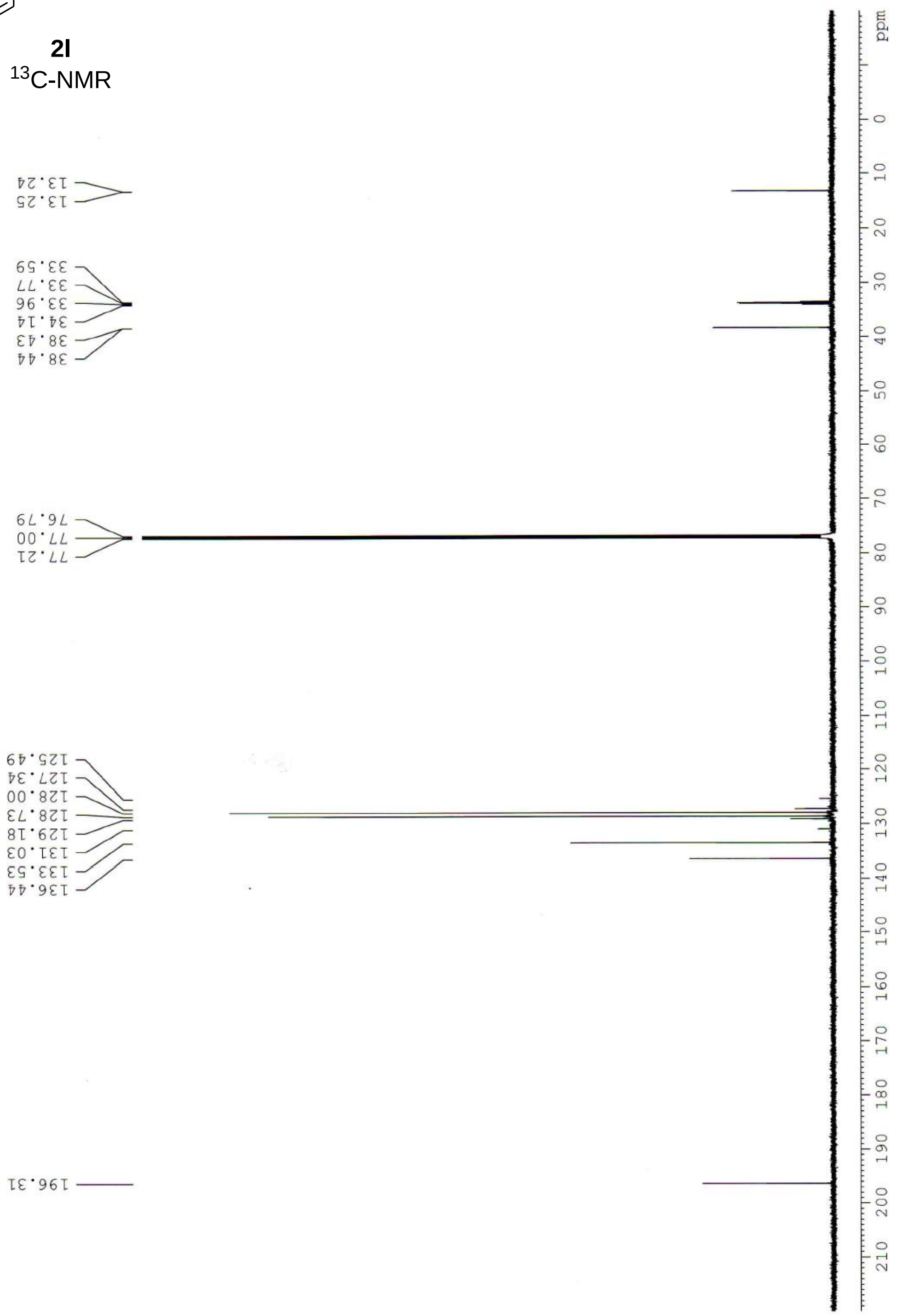
13.24
13.25
33.59
33.77
33.96
34.14
38.43
38.44
76.79
77.00
77.21
125.49
127.34
128.00
128.73
129.18
131.03
133.53
136.44
196.31
ppm
